# Genetic dissection of nutrient element enrichment in *Saccharum officinarum* L. for deciphering the food health

**DOI:** 10.3389/fpls.2025.1649792

**Published:** 2025-12-04

**Authors:** Lina Fan, Zaid Chachar, Ming Chen, Yucong Li, Jiarui Chen, Huiyi He, Jiakun Wen, Saira Gul, Ruiqiang Lai, Yongwen Qi

**Affiliations:** 1 College of Agriculture and Biology, Zhongkai University of Agriculture and Engineering, Guangzhou, Guangdong, China; 2 Institute of Nanfan and Seed Industry, Guangdong Academy of Science, Guangzhou, Guangdong, China; 3 Shenzhen Wemed Medical Equipment Co., Ltd., Shenzhen, China

**Keywords:** *Saccharum officinarum*, nutrient element, GWAS, genetic dissection, food health

## Abstract

**Introduction:**

Sugarcane is a critical global crop, primarily used for sugar production, with applications spanning the food, industrial, and pharmaceutical sectors. Improving nutrient efficiency in sugarcane varieties is essential to enhance yield, ensure sugar security, and improve nutritional value.

**Methods:**

This study analyzed 109 sugarcane stem segment samples to assess major (magnesium, calcium, phosphorus) and trace (manganese, iron, copper) nutrient content, along with SNP genotyping data. Two multi-model Genome-Wide Association Study (GWAS) approaches—MLM_Q+Kinship and MLM_PCA+Kinship—were employed to identify SNPs associated with nutritional traits.

**Results:**

Under a P-value threshold of <10⁻⁴, the MLM_Q+Kinship approach identified SNPs linked to several traits, including total sugar (4), nitrogen (7), phosphorus (179), potassium (4), sulfur (41), magnesium (26), calcium (20), and silicon (66). Similar results were obtained with MLM_PCA+Kinship. A stricter threshold (P < 1.53×10⁻⁶) yielded 59 reliable "Peak" SNP loci, which explained over 15% of phenotypic variance. Notably, SNPs 3B_33126337 and 5C_64683842 were strongly associated with magnesium, calcium, and iron utilization. Genotypic analysis revealed superior nutrient accumulation in genotypes TT (3B_33126337) and AA/AG (2D_29369331, 3B_14919731). Bioinformatics analysis identified 12 candidate genes involved in magnesium, calcium, and manganese metabolism, linked to pyruvate metabolism, purine metabolism, and glycolysis/gluconeogenesis pathways. RT-qPCR analysis confirmed significant expression differences in three genes (Sspon.003B0015150, Sspon.002D0013560, and Sspon.003B0007030) between genotypes, suggesting their potential as key targets.

**Conclusion:**

These findings provide valuable genetic markers and candidate genes for the development of nutrient-efficient, high-yield sugarcane varieties, contributing to advancements in both agriculture and nutrition.

## Introduction

Sugarcane (*Saccharum officinarum*) is a globally significant crop, primarily cultivated for its high sucrose content, which is utilized for sugar production, biofuel, and other industrial purposes (Luo et al., 2023). However, productivity and quality are often constrained by the efficiency of nutrient utilization, which is a critical factor for improving yield and nutritional content. Understanding the genetic basis of nutrient utilization in sugarcane is essential for developing varieties that are both high-yielding and nutritionally optimized. Recent advances in genomics have provided new insights into the molecular mechanisms underlying nutrient uptake and accumulation in crops, including sugarcane (Adhikari et al., 2022).

Genetic studies, such as Genome-Wide Association Studies (GWAS), have become indispensable tools for identifying markers associated with key agronomic traits. These studies help pinpoint specific loci that are responsible for desirable traits, such as nutrient efficiency. GWAS in crops, including sugarcane, has revealed the genetic underpinnings of traits such as disease resistance, drought tolerance, and nutrient use efficiency (Ahmad and Ming, 2024). However, the genetic factors contributing to the uptake and accumulation of specific nutrients, such as magnesium, calcium, and manganese, have not been thoroughly explored in sugarcane, thus limiting the potential for breeding nutrient-efficient varieties.

The identification of reliable single-nucleotide polymorphisms (SNPs) associated with nutrient efficiency can significantly enhance the breeding of sugarcane varieties with superior nutrient profiles. Previous studies have shown that the use of population structure and kinship coefficients in GWAS can help minimize false positives and improve the accuracy of identifying marker-trait associations (Sandhu et al., 2022). Furthermore, the integration of multi-model approaches, such as MLM_Q + Kinship and MLM_PCA + Kinship, has demonstrated promise in identifying robust SNP loci associated with important agronomic traits in many plants, such as maize (Zhu et al., 2023) and tobacco (Lai et al., 2024), and in sugarcane (Li, 2024) also achieved excellent results by using the MLM_Q + Kinship model. Despite the growing interest in genomics, there remains a gap in the systematic identification and validation of SNP markers linked to nutrient utilization in sugarcane.

In this study, we aimed to identify SNP loci associated with key nutrient elements in the juice of sugarcane varieties using a genome-wide association approach. Specifically, we focused on macronutrients (sugar content, nitrogen, phosphorus, potassium, sulfur, magnesium, calcium, and silicon) and micronutrients (iron, manganese, zinc, copper, molybdenum, and germanium) that play crucial roles in sugarcane growth and development. By employing rigorous screening methods, we identified reliable SNP markers and superior genotypes associated with efficient nutrient utilization. In addition, bioinformatics analyses were conducted to predict the molecular functions of candidate genes located near these SNPs. Reverse Transcription quantitative Polymerase Chain Reaction (RT-qPCR) was used to analyze the relative expression levels of candidate genes between materials with and without excellent genotypes to further identify candidate genes that deserve priority attention. The ultimate goal of this research is to contribute to the development of nutrient-efficient sugarcane varieties for deciphering food health, thereby enhancing crop yield and nutritional quality while promoting sustainable agricultural practices.

## 2 Materials and methods

### 2.1 Plant materials and phenotypic data

The test population consisted of 109 sugarcane materials (Lai et al., 2024). They had a wide range of origins, covering countries such as China, the United States, India, Australia, the Philippines, Mauritius, Brazil, France, Cuba and Japan, etc. The breeding years spanned from 1930 to 2010, with 9 of them having unknown breeding years. Further details of the materials for Co281, Co419, Neijiang-57-416, Yuetang65_1279 and Ganzhe65-542, further details of the materials are shown in **Supplementary Table S1**.

In 2022, this population was planted at the Wengyuan Experimental Base (24°17′N, 113°56′E). Each material was planted in three replicates side-by-side. During planting, each sugarcane was kept with a single bud, with a row spacing of 1.1 m and a plant spacing of 25 cm. Sixteen plants of each material were planted in each replicate, and pest, disease, and weed control were performed according to normal field management measures. At the maturity stage of the test materials, 10 middle stem segments of each material with good growth and consistent growth were selected. Each stem segment had approximately 4–5 nodes and was crushed, juiced, and mixed. After impurity removal, the sugarcane juice was digested with 65% concentrated nitric acid in a digestion instrument according to the method of (Zhu et al., 2023). The macronutrients phosphorus (P), potassium (K), sulfur (S), magnesium (Mg), calcium (Ca), and silicon (Si) and micronutrients iron (Fe), manganese (Mn), zinc (Zn), copper (Cu), molybdenum (Mo), and germanium (Ge) were determined by ICP–MS. The macronutrient nitrogen (N) was determined by the Kjeldahl method (Hejcman et al., 2013), and the total sugar content of the sugarcane juice was determined by BRIX METER (BM - 04 Nohawk) (Ahmed et al., 2022).

Rang, Mean, standard deviation, Kurtosis, Skewness, and coefficient of variation were statistically analyzed using IBM SPSS Statistics 27.0 software. The best linear unbiased prediction (BLUP) was calculated using the lme4 package (https://github.com/lme4) in R_4.0 (https://www.r-project.org/) with a mixed linear model. A normal distribution plot was generated using the ‘NORMDIST’ function (Stöckl and Thienpont, 2008; Lai et al., 2024).

### 2.2 Quality control and LD analysis of SNP genotyping data

The raw data of SNP genotyping for 109 materials were obtained from a previous study (Li et al., 2024), and then referred to genome AP85-441 (*Saccharum* sp*ontaneum*) (Chen et al., 2024) for SNP extraction had. Subsequently, plink 1.90 software (https://www.cog-genomics.org/plink2/) was utilized to conduct quality control of the data. Loci with a minor allele frequency less than 0.05 and a missing rate greater than 5% were removed. Additionally, the Plink parameter -indep-pairwise 100 50 0.2, was set to reduce the redundant SNP loci caused by linkage disequilibrium (LD). Eventually, a total of 653019 high-quality SNPs were screened.

The PopLDdecay software (https://github.com/BGI-shenzhen/PopLDdecay.git) was employed to calculate the average linkage disequilibrium decay distance (LD) of the associated population. The ggplot2 package (https://ggplot2.tidyverse.org/.

) of R_4.0 software was used to draw the LD decay plot.

### 2.3 Kinship, population structure, PCA analysis

The kinship matrix (Kinship) was calculated using TASSEL 5.0 software (https://www.maizegenetics.net/tassel), and a kinship heatmap was plotted with the ComplexHeatmap package of R_4.0 software (https://github.com/jokergoo/ComplexHeatmap).

Population structure analysis was carried out using Admixture 1.3.0 software (https://dalexander.github.io/admixture/download.html). The K value was sequentially set from 1 to 12 to obtain the CV. error for each K value, and the minimum CV error was taken as the optimal clustering threshold K. Subsequently, the distribution map of the optimal K value was drawn using the ggplot2 package of R_4.0, and the Q matrix (Q) heatmap of the population structure was generated using the graphics package.

The principal component score matrix (PCA) of the samples was obtained through PCA analysis using the Plink 1.90 software. The samples were grouped according to the classification results of the population structure, and a PCA plot was drawn using the ggplot2 package of R_4.0 software.

### 2.4 GWAS, phenotypic effect value, and identification of superior genotypes

In the GWAS, PCA + Kinship and Q + Kinship were used as covariates, and the analysis was carried out based on a mixed linear model (MLM), including the MLM_Q+Kinship and MLM_PCA+Kinship models. GWAS results were visualized as Manhattan plots using the CMplot package (https://github.com/YinLiLin/CMplot) in R_4.0. The judgment of the availability of association analysis model was used the QQ plots and the lambda value (λ-value = median(chi2)/0.456) using R_4.0 (Paternoster et al., 2011; Lai et al., 2024). The significant association between SNPs and sugarcane element content was determined according to loose (P-value < 1×10^-4^) and strict (P-value < 1/653019 ≈ 1.53×10^-6^) thresholds.

The phenotypic effect value analysis referred to the methods in previous studies (Zhu et al., 2023; Lai et al., 2024). The calculation formula is Fi = ∑Sij/ni − ∑Ny/ny. where Fi represents the phenotypic effect value of genotype i, ∑Sij/ni represents the average phenotypic value of all materials with genotype i, and ∑Ny/ny represents the average phenotypic value of all materials. If Fi > 0, it indicates that genotype i tends to increase the phenotypic value, and it is recommended that this locus is a superior genotype. If Fi < 0, it means that the phenotypic value of materials with genotype i tends to be low, and this genotype is the corresponding genotype. SPSS 27.0 software was used to understand the significant differences (P-value < 0.05) in phenotype values between different genotypes via analysis of variance (Zhu et al., 2023).

### 2.5 Bioinformatics analysis and RT-qPCR

Protein-protein interactions were analyzed using the STRING software (https://cn.string-db.org/) (Szklarczyk et al., 2023). The molecular weight, isoelectric point, and hydrophilicity of the proteins were calculated using the corresponding subroutines in the online tool (https://www.detaibio.com/sms2/). Amino acid sequence alignment analysis was performed using DNAman software. GO and KEGG analyses were carried out using KOBAS (http://bioinfo.org/kobas/annotate/) software. The BWRPSB software (https://www.ncbi.nlm.nih.gov/Structure/bwrpsb/bwrpsb.cgi) was employed to predict the conserved domains of protein families, while the cis-acting element prediction of the 2000 bp upstream of the gene + 1 region was conducted using PLANTCARE software (http://bioinformatics.psb.ugent.be/webtools/plantcare/html/). Finally, the Visualize NCBI CDD DomainPattern function of the tbtools software was used for domain visualization, and the simple biosequence viewer function was used for the visualization of cis-acting elements. Subcellular localization prediction of proteins was performed using WoLF PSORT (https://wolfpsort.hgc.jp/) using PSORT features and traditional PSORTII prediction.

Sugarcane with good growth at the jointing stage on July 30, 2024, was selected for total RNA extraction and reverse transcribed into cDNA from the experimental field in Fusui County, Chongzuo City, Guangxi Zhuang Autonomous Region (22°31′N, 107°31′E). Subsequently, we obtained homologous genes from (Chen et al., 2024), compared their CDS sequences, and then selected specific sequences. Finally, gene-specific primers were designed for RT-qPCR, with GAPDH (ID: EF189713) as an internal control. Three replicate experiments were performed, and variance analysis (P < 0.05) was performed.

## 3 Result

### 3.1 Phenotypic analysis

Identifying the enrichment levels of nutrient elements in sugarcane is conducive to optimizing the growth and development of sugarcane, and more importantly, to providing a high-quality source of nutrition for humans. Therefore, a statistical analysis was conducted on the phenotypes of 14 essential nutrient elements in 109 sugarcane samples, including macronutrients (total sugar, nitrogen, phosphorus, potassium, sulfur, magnesium, calcium, and silicon), as well as essential micronutrients (iron, manganese, zinc, copper, molybdenum, and germanium). The results (**Table 1**) showed that the mean±standard deviation (SD) of the contents of total sugar, nitrogen, phosphorus, potassium, sulfur, magnesium, calcium, silicon, iron, manganese, zinc, copper, molybdenum, and germanium were 15.50096±3.56395%, 0.76626±0.23496 mg·g^-^¹, 86.36581±53.92837 mg·g^-^¹, 0.84398±0.00227 mg·g^-^¹, 779.67129±577.55969 mg·g^-^¹, 267.34549±206.98324 mg·g^-^¹, 760.34603±550.36127 mg·g^-^¹, 556.12793±313.47098 mg·g^-^¹, 44.79544±17.80614 mg·g^-^¹, 13.63706±10.01638 mg·g^-^¹, 14.28088±6.29195 mg·g^-^¹, 1.40448±0.47391 mg·g^-^¹, 0.45102±0.36370 mg·g^-^¹, and 0.00635±0.00349 mg·g^-^¹, respectively. Their distribution ranges (**Table 1**) were 7.50500 - 26.05000%, 0.33940 - 1.44687 mg·g^-^¹, 22.71676 - 334.79358 mg·g^-^¹, 0.83800 - 0.85080 mg·g^-^¹, 166.54347 - 2979.94242 mg·g^-^¹, 30.46414 - 918.48547 mg·g^-^¹, 29.42590 - 2356.33701 mg·g^-^¹, 51.37524 - 1612.09199 mg·g^-^¹, 16.51630 - 104.63089 mg·g^-^¹, 3.61582 - 64.71526 mg·g^-^¹, 4.10109 - 35.81823 mg·g^-^¹, 0.64158 - 2.93846 mg·g^-^¹, 0.00514 - 2.17485 mg·g^-^¹, and 0.00034 - 0.02368 mg·g^-^¹, respectively. The kurtosis (**Table 1**) were -0.23606, -0.23606, 5.81324, 0.56981, 1.78800, 0.85156, 0.00800, 2.25367, 1.24387, 9.18879, 0.35818, 0.75839, 5.20337, and 4.25572, respectively. The skewness (**Table 1**) were 0.05090, 0.63357, 1.98976, 0.11639, 1.31707, 1.30643, 1.00700, 1.52387, 0.96800, 2.76569, 0.61023, 0.93605, 1.99044, and 1.29308, respectively. The coefficients of variation (**Table 1**) were 22.99181%, 30.66340%, 62.44181%, 0.26872%, 74.07733%, 77.42163%, 72.38300%, 56.36670%, 39.74989%, 73.44966%, 44.05856%, 33.74250%, 80.63818%, and 54.99352%, respectively.

**Table 1:** Statistical analysis of fourteen nutritional elements.

Type	Element	Rang	Mean±SD	Kurtosis	Skewness	CV/%
macronutrients	TS	7.50500~26.05000%	15.50096±3.56395%	-0.23606	0.05090	22.99181
	N	0.33940 - 1.44687 mg·g^-^¹	0.76626±0.23496 mg·g^-^¹	-0.23606	0.63357	30.66340
	P	22.71676 - 334.79358 mg·g^-^¹	86.36581±53.92837 mg·g^-^¹	5.81324	1.98976	62.44181
	K	0.83800 - 0.85080 mg·g^-^¹	0.84398±0.00227 mg·g^-^¹	0.56981	0.11639	0.26872
	S	166.54347 - 2979.94242 mg·g^-^¹	779.67129±577.55969 mg·g^-^¹	1.78800	1.31707	74.07733
	Mg	30.46414 - 918.48547 mg·g^-^¹	267.34549±206.98324 mg·g^-^¹	0.85156	1.30643	77.42163
	Ca	29.42590 - 2356.33701 mg·g^-^¹	760.34603±550.36127 mg·g^-^¹	0.00800	1.00700	72.38300
	Si	51.37524 - 1612.09199 mg·g^-^¹	556.12793±313.47098 mg·g^-^¹	2.25367	1.52387	56.36670
micronutrients	Fe	16.51630 - 104.63089 mg·g^-^¹	44.79544±17.80614 mg·g^-^¹	1.24387	0.96800	39.74989
	Mn	3.61582 - 64.71526 mg·g^-^¹	13.63706±10.01638 mg·g^-^¹	9.18879	2.76569	73.44966
	Zn	4.10109 - 35.81823 mg·g^-^¹	14.28088±6.29195 mg·g^-^¹	0.35818	0.61023	44.05856
	Cu	0.64158 - 2.93846 mg·g^-^¹	1.40448±0.47391 mg·g^-^¹	0.75839	0.93605	33.74250
	Mo	0.00514 - 2.17485 mg·g^-^¹	0.45102±0.36370 mg·g^-^¹	5.20337	1.99044	80.63818
	Ge	0.00034 - 0.02368 mg·g^-^¹	0.00635±0.00349 mg·g^-^¹	4.25572	1.29308	54.99352

TS, N, P, K, S, Mg, Ca, Si, Fe, Mn, Zn, Cu, Mo, and Ge represent total sugar, nitrogen, phosphorus, potassium, sulfur, magnesium, calcium, silicon, iron, manganese, zinc, copper, molybdenum, and germanium, respectively.

These results indicated that the content variations of eight elements, namely phosphorus, sulfur, magnesium, calcium, silicon, manganese, molybdenum, and germanium, were relatively large, with their coefficients of variation all greater than 50% (**Table 1**). In contrast, the content variations of the other six elements, namely, total sugar, nitrogen, potassium, iron, zinc, and copper, were relatively small, with an average coefficient of variation of 29% (**Table 1**). However, all 14 nutrient elements exhibited variation. The kurtosis and skewness of most nutrient elements were close to zero (**Table 1**), showing a right-skewed trend. Only the kurtosis of phosphorus, manganese, molybdenum, and germanium were relatively large (**Table 1**), but they all showed an approximately normal distribution (**Supplementary Figure S1**; **S2**). This suggests that there are some potentially high-quality materials in this population that can be screened as basic materials, which is beneficial for exploring gene loci related to these nutrient elements.

**Figure 1:**
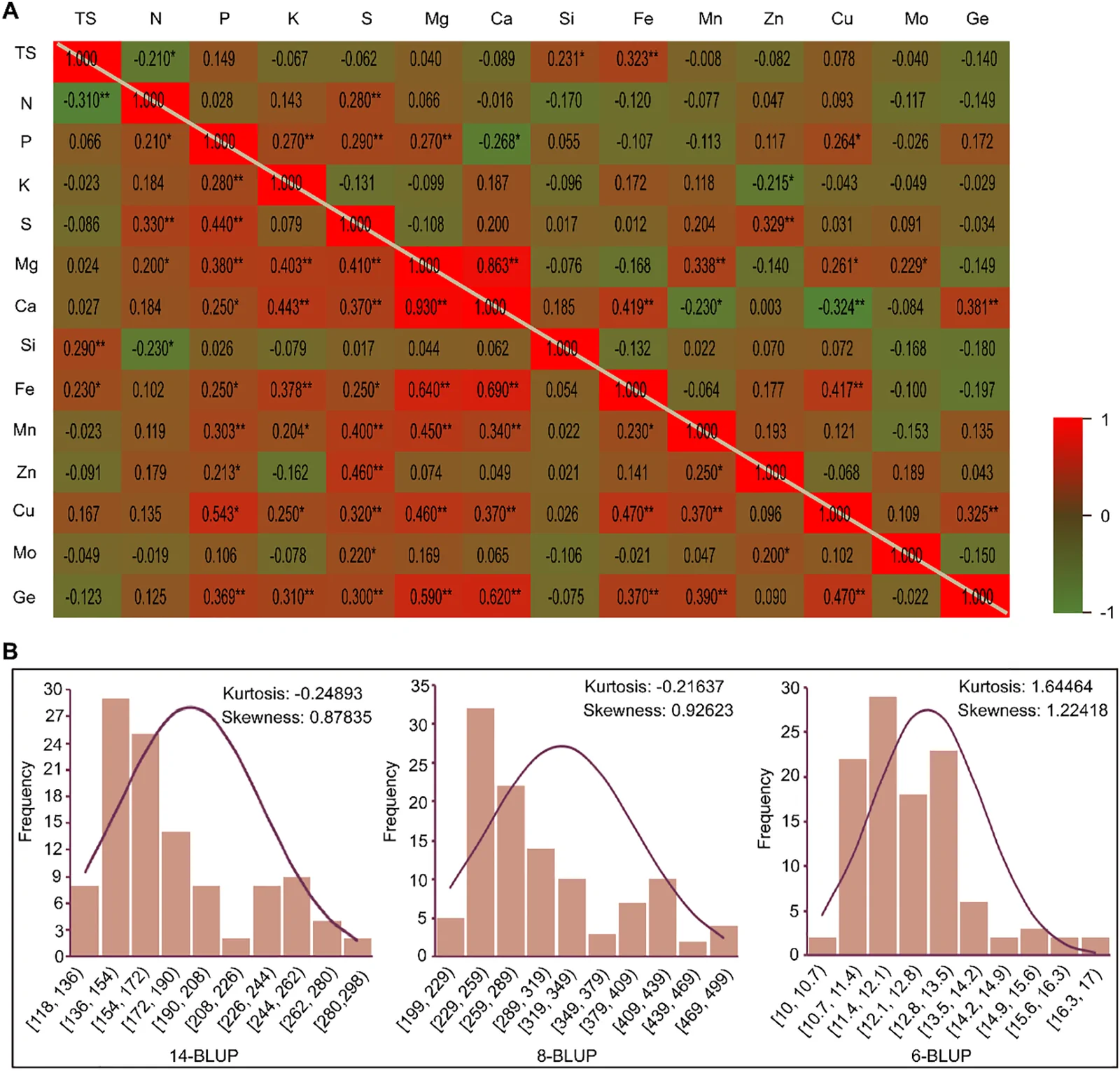
Correlation analysis of 14 nutrient elements and the distribution of the value of best linear unbiased prediction. **(A)** Simple Correlation Analysis (lower left corner of the yellow line) and Partial Correlation Analysis (upper right corner of the yellow line) among the 14 nutrient elements. TS: Total Sugar; N: Nitrogen; P: Phosphorus; K: Potassium; S: Sulfur; Mg: Magnesium; Ca: Calcium; Si: Silicon; Fe: Iron; Mn: Manganese; Zn: Zinc; Cu: Copper; Mo: Molybdenum; Ge: Germanium. “*” indicates a significant correlation (P < 0.05); “**” indicates an extremely significant correlation (P < 0.01). **(B)** Distribution of the value of best linear unbiased prediction for 14 nutrient elements (14-BLUP), 8 macronutrient elements (8-BLUP), and 6 micronutrient elements (6-BLUP). Among them, TS, N, P, K, S, Mg, Ca, and Si are macronutrient elements; Fe, Mn, Zn, Cu, Mo, and Ge are micronutrient elements.

It is worth mentioning that the selection and breeding of materials rich in multiple nutrient elements and with an appropriate nutrient structure are of great significance for promoting the breeding process of high-yield and nutritious sugarcane. Therefore, this study conducted Pearson correlation and partial correlation analyses on the 14 nutrient elements. The results showed that there were a total of 98 pairs of combinations among the 14 nutrient elements, among which 47 pairs had significant correlations (**Figure 1A**). The number of significant correlations between each element and the other elements ranged from 2 to 10 (**Figure 1A**). Since partial correlation analysis can essentially reflect the relationship between two traits on the basis of correlation analysis, partial correlation analysis was also carried out on the 14 nutrient elements. The results revealed that only 21 pairs had significant correlations, and the number of significant correlations between each element and the other elements ranged from 1 to 6 (**Figure 1A**). Interestingly, the results of partial correlation analysis and Pearson correlation analysis (**Figure 1A**) showed that the absolute value of the partial correlation coefficient between iron and total sugar (partial correlation coefficient 0.323** > Pearson correlation coefficient 0.230*), between zinc and potassium (| -0.215*| > | -0.162|), and between molybdenum and manganese (0.229* > 0.169) was greater than that of the Pearson correlation coefficient, indicating that these three combinations had a good linear relationship. However, the correlations between some elements presented the opposite situation (**Figure 1A**). For example, the Pearson correlation between calcium and phosphorus was a positive correlation of 0.250*, but it was a negative correlation of -0.215* in the partial correlation analysis; and the Pearson correlation between manganese and calcium was a positive correlation of 0.340**, while it was a negative correlation of -0.230* in the partial correlation analysis. This result indicates that the absorption and utilization of different elements by plants are affected by multiple factors and are complex. Clarifying the relationships between them is crucial for guiding fertilization and promoting high-efficiency crop nutrition.

In addition, the breeding value can consider comprehensive genetic information, reduce the influence of environmental factors, and by predicting the breeding value of the overall phenotypic data of different nutrient elements, the situation of the breeding value of materials when they contain multiple nutrient elements can be obtained. Therefore, in this study, the phenotypic data of 14 nutrient elements, 8 macronutrients, and 6 micronutrients were used for breeding value prediction, namely the value of best linear unbiased prediction (BLUP), which were recorded as 14-BLUP, 8-BLUP, and 6-BLUP, respectively. The results showed that their kurtosis (skewness) were -0.24893 (0.87835), -0.21637 (0.89623), and 1.64464 (1.22418), respectively, also showing an approximately normal distribution (**Figure 1B**). Among them, when the 14-BLUP was arranged in descending order, the top 10 materials were C89-51 (288.84587), M142-59 (280.08386), Yacheng-84-153 (279.72953), CP72-2086 (277.61687), Q208 (271.67914), Co281 (265.88647), yue83_271 (254.52622), Chuan-83-181 (253.99698), HOTH344 (253.96588), Yacheng-93-26 (253.06481); when the 8-BLUP was arranged in descending order, the top 10 materials were C89-51 (496.79533), Yacheng-84-153 (481.91189), CP72-2086 (478.26707), M142-59 (475.20906), Q208 (468.11197), Co281 (457.52028), yue83_271 (436.88720), Chuan-83-181 (436.82559), HOTH344 (434.86203), Yacheng-93-26 (434.22801); and when the 6-BLUP was arranged in descending order, the top 10 materials were C529-50 (16.70545), yue83_271 (16.42863), Dezhe-03-83 (16.21221), YT-60_YT-03-393 (15.75246), CP72-1210 (15.57346), KeWu (15.17350), zhan74-141 (15.00150), HOTH344 (14.40163), Chuan-73-219 (14.36101), C89-51 (14.17530). Among them, the results of material screening based on the breeding value of macronutrients and secondary macronutrients (8-BLUP) were consistent with those of the breeding value of macronutrients, secondary macronutrients, and micronutrients (14-BLUP), but differed significantly from the results of the breeding value of micronutrients (6-BLUP). Interestingly, all three methods identified material C89-51, which can be regarded as a potential optimization material.

### 3.2 Analysis of population genetic diversity

**Figure 2:**
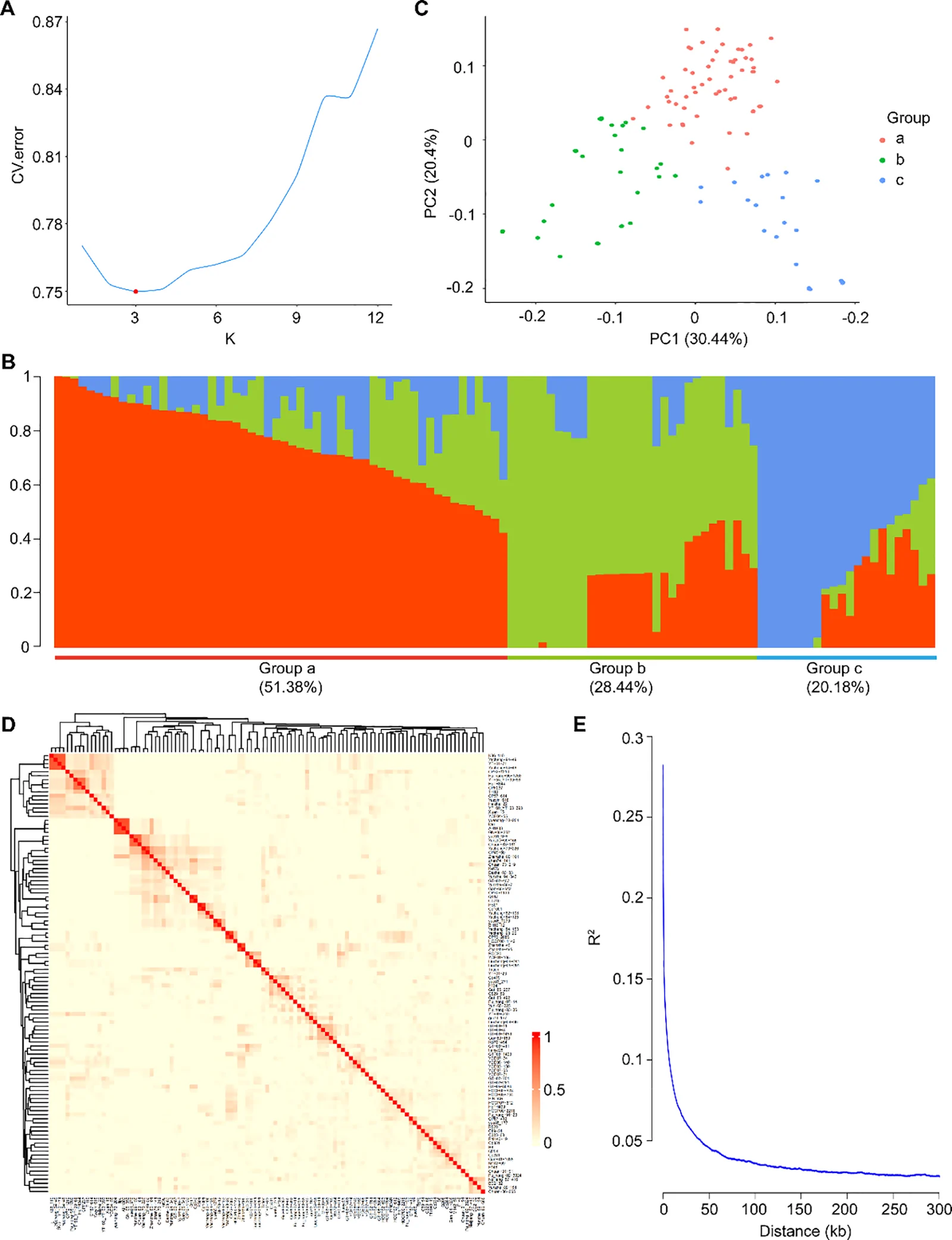
Population structure, principal component analysis, kinship, and linkage disequilibrium analysis. **(A)** Determination of the optimal number of subpopulations based on the CV error calculated using Admixture 1.3.0 Software. The optimal K value, representing the number of subpopulations, is determined as that corresponding to the minimum CV error. **(B)** Classification of 109 materials based on the results from **(A)** using the Q value. These subpopulations were designated as Group a (red), Group b (green), and Group c (blue). **(C)** Principal Component Analysis (PCA). The PCA plot distinguishes Group a (red), Group b (green), and Group c (blue) based on their respective positions. These group assignments were consistent with the subpopulation classifications presented in **(B)**. **(D)** Kinship coefficient analysis. The kinship coefficients among the materials were calculated using TASSEL 5.0 software and visualized using the ComplexHeatmap package of R_4.0 software. **(E)** Linkage disequilibrium (LD) analysis. LD decay is evaluated by computing R2 values using PopLDdecay software. The LD decay plot is generated using the ggplot2 package of R_4.0 software.

Genetic diversity is an important component of biodiversity, and its research is conducive to the protection, development, and utilization of germplasm resources. Therefore, in this study, population structure analysis, Principal Component Analysis (PCA), kinship coefficient analysis, and linkage disequilibrium analysis were conducted on the SNP genotyping results of 109 sugarcane populations. The results of the population structure analysis showed that this population had three lineages (**Figure 2A**). Therefore, this population was classified into three subclasses (**Figure 2B**; **Supplementary Table S2**): group a, group b, and group c. The proportions of materials in each group relative to the total number of materials were 51.38%, 28.44%, and 20.18%, respectively. It is worth noting that based on PCA analysis (**Figure 2C**), it was also found that the classification of these materials was consistent with the results of the population structure analysis. Thus, the reliability of the Q value or PCA value identified by population structure analysis as the correction coefficient for subsequent association analysis was high.

In addition, kinship is also an important indicator reflecting the genetic diversity among materials and can serve as an important correction coefficient for association analysis, which is beneficial for reducing the probability of false-positive associations. Therefore, in this study, the kinship coefficients between each pair of 109 materials (**Figure 2D**) for a total of 5886 combinations were calculated. The results showed that the range of kinship coefficients was 0–0.9325. Among them, 5861 combinations had kinship coefficients less than 0.5, accounting for 99.58% of the total combinations. The number of combinations with kinship coefficients less than 0.05 was 5007, accounting for 85.07% of the total combinations, and the number of combinations with kinship coefficients equal to 0 was 4042, accounting for 68.67% of the total combinations. These results indicate that most of the tested germplasms had weak or no kinship relationships, and the genetic diversity of the selected germplasms was relatively rich.

Furthermore, in linkage disequilibrium analysis, the R^2^ value reflects the correlation coefficient between two loci and is closely related to the effectiveness of the association analysis. Therefore, in this study, the R^2^ values between each pair of the 986,879 screened SNPs were calculated. The results showed that the range of R^2^ values was 0.0265 to 0.2915, with an average of 0.0388 with low linkage. When the LD decay dropped to 0.05, the LD decay distance was ~37kb, which is a relatively small decay distance. This indicates that there were large genetic differences among the selected materials and rich combinations of different gene loci, which is beneficial for subsequent association analysis.

### 3.3 Genome-wide association analysis and screening reliable SNP loci

Using the corrected coefficients Q values (or PCA values) of each individual and the kinship coefficients obtained from population structure analysis as covariates for correction is an effective way to avoid false positives in the association analysis between genotypes and phenotypes (Yu et al., 2006). Therefore, in this study, based on the population structure correction coefficient Q values or PCA values of SNP genotyping data and the kinship coefficient Kinship, the association efficiencies of two association models, MLM_Q + Kinship (**Supplementary Figure S3A, B**) and MLM_PCA + Kinship (**Supplementary Figure S3C, D**), were evaluated for 14 nutrient elements in the juice of 109 sugarcane varieties, their breeding values (14-BLUP), breeding values of macronutrients (8-BLUP), and breeding values of micronutrients (6-BLUP). The results showed that both models could effectively identify SNP loci related to the 14 nutrient elements and their breeding values. Their Lambda values were approximately equal to 1 (**Supplementary Figure S3**), with a high degree of coincidence, and could effectively reduce spurious associations (Lai et al., 2024). Consequently, a certain number of SNP loci were identified under both less stringent conditions (P < 1×10^-4^) and strict conditions (P < 1/986879 ≈ 1.53×10^-6^) (**Supplementary Figure S3A, C**; **Supplementary Table S3**, **S4**).

Among them, based on the MLM_Q + Kinship model (P < 1.00×10^-4^) (**Supplementary Table S3**), 4, 7, 179, 4, 41, 26, 20, 66, 37, 409, 14, 27, 299, 142, 21, 19, and 49 SNPs were identified for total sugar, nitrogen, phosphorus, potassium, sulfur, magnesium, calcium, silicon, iron, manganese, zinc, copper, molybdenum, germanium, 14-BLUP, 8-BLUP, and 6-BLUP, respectively. Based on the MLM_PCA + Kinship model (P < 1.00×10^-4^) (**Supplementary Table S4**), 4, 7, 181, 5, 42, 25, 22, 64, 40, 406, 14, 28, 297, 139, 21, 20, and 51 SNPs were identified for total sugar, nitrogen, phosphorus, potassium, sulfur, magnesium, calcium, silicon, iron, manganese, zinc, copper, molybdenum, germanium, 14-BLUP, 8-BLUP, and 6-BLUP, respectively.

**Figure 3:**
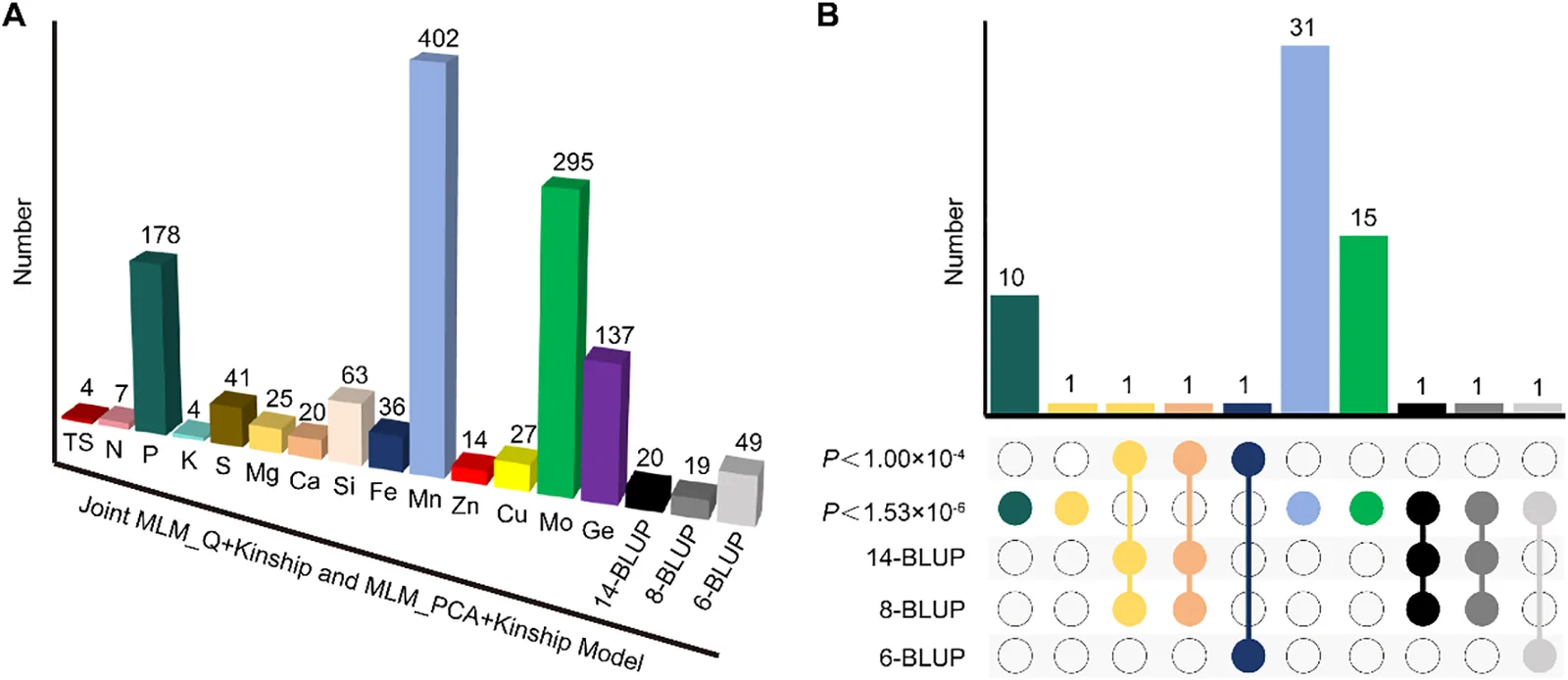
The number of Peak SNPs related to 14 nutrient elements and BLUP. **(A)** The number of SNPs (P-value < 1.00×10^-4^) identified by both the MLM_Q + Kinship and MLM_PCA + Kinship models. **(B)** The number of Peak SNPs identified under the high threshold condition (P-value < 1.53×10^-6^) or the number of Peak SNPs identified under the BLUP condition (P-value < 1.53×10^-6^) and also identified as Peak SNPs (P-value < 1.00×10^-4^) in a single nutrient element. TS (dark red): Total Sugar; N (scarlet): Nitrogen; P (deep cornflower blue): Phosphorus; K (light cornflower blue): Potassium; S (dark orange): Sulfur; Mg (light orange): Magnesium; Ca (dark chocolate yellow): Calcium; Si (light chocolate yellow): Silicon; Fe (dark steel blue): Iron; Mn (light steel blue): Manganese; Zn (red): Zinc; Cu (yellow): Copper; Mo (green): Molybdenum; Ge (purple): Germanium; 14-BLUP (black): the value of best linear unbiased prediction of 14 nutrient elements; 8-BLUP (light black): the value of best linear unbiased prediction of 8 macronutrient elements; 6-BLUP (light gray): the value of best linear unbiased prediction of 6 micronutrient elements.

Finally, the two models (P < 1.00×10^-4^) (**Figure 3A**; **Supplementary Table S5**) jointly identified 4, 7, 175, 4, 41, 23, 20, 62, 36, 402, 14, 27, 295, 137, 20, 19, and 49 Peak SNPs for total sugar, nitrogen, phosphorus, potassium, sulfur, magnesium, calcium, silicon, iron, manganese, zinc, copper, molybdenum, germanium, 14-BLUP, 8-BLUP, and 6-BLUP, respectively. The phenotypic variance explained (PVE) by these SNPs was higher than 15% (**Supplementary Table S5**) and since these SNPs were identified in both models, they can be regarded as important Peak SNPs (Ikram et al., 2022). In this study, to further improve the reliability of Peak SNPs, we used a more stringent screening threshold (P < 1.53×10^-6^) to screen the SNPs identified by both models. The results showed that for phosphorus, magnesium, manganese, molybdenum, 14-BLUP, 8-BLUP, and 6-BLUP, 10, 1, 31, 15, 1, 1, and 1 SNPs were identified, respectively, and their PVE phenotypic variance explained was greater than 29% (**Figure 3B**; **Supplementary Table S6**). It is worth noting that for both magnesium and calcium, there was 1 SNP, namely 3B_33126337 (P < 1.00×10^-4^), which was also identified in 14-BLUP (P < 1.53×10^-6^) and 8-BLUP (P < 1.53×10^-6^) (**Figure 3B**; **Supplementary Table S5**, **S6**). For iron, there was also 1 SNP, namely 5C_64683842 (P < 1.00×10^-4^), which was also identified in 6-BLUP (P < 1.53×10^-6^) (**Figure 3B**; **Supplementary Table S5**, **S6**). In summary, a total of 59 Peak SNPs (**Figure 3B**; **Supplementary Table S6**) that meet the strict screening threshold and have a high phenotypic variance explained rate were relatively reliable SNP loci. Notably, the SNP locus 3B_33126337 is highly likely to play an important role in the utilization process of the 14 nutrient elements, especially macronutrients such as magnesium and calcium; while the SNP locus 5C_64683842 is highly likely to play an important role in the utilization process of the 6 micronutrients, such as iron. Therefore, subsequent analyses of the two loci (**Supplementary Table S7**), 3B_33126337 and 5C_64683842, would also focus on magnesium, calcium and iron elements, respectively.

### 3.4 Phenotypic effect analysis, identification of superior genotypes, and screening of nutrient-efficient candidates

**Figure 4:**
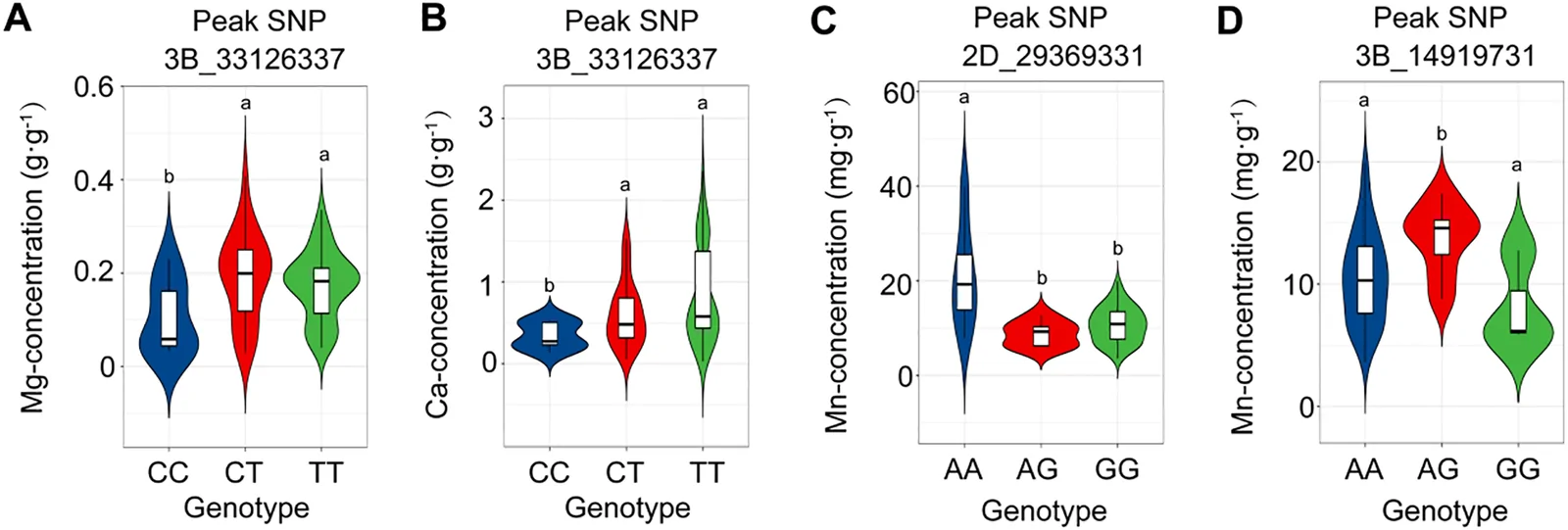
Violin plots of phenotypic values of different genotypes at the Peak SNP. **(A)** Analysis of variance for the phenotypic magnesium content of different genotypes at the 3B_33126337 SNP locus. **(B)** Analysis of variance for the phenotypic calcium content of different genotypes at the 3B_33126337 SNP locus. **(C)** Analysis of variance for the phenotypic manganese content of different genotypes at the 2D_29369331 SNP locus. **(D)** Analysis of variance for the phenotypic manganese content of different genotypes at the 3B_14919731 SNP locus. Different letters on the violin plots indicate significant differences (P < 0.05), while the same letters indicate no significant differences.

To investigate the impact of the 59 reliable SNP loci on phenotypic and breeding values, this study conducted a phenotypic effect analysis for their different genotypes. The results revealed that all 59 SNP loci exhibited three genotypes, with superior genotypes demonstrating a tendency to absorb and accumulate the corresponding nutrients (**Supplementary Table S7**). Consequently, materials with different genotypes for these 59 SNP loci were classified, and variance analysis was performed on their phenotypic values. Subsequent significant phenotypic differences between the superior genotypes of the three SNPs and their corresponding relative genotypes were identified (**Figures 4A-D**; **Supplementary Table S7**), including 3 B _33126337 associated with Mg and Ca, and 2D_29369331 and 3 B _14919731 both associated with Mn.

At the 3B_33126337 locus, materials with the TT genotype had the highest positive effect values (>0) for Mg and Ca, averaging 26.09966 mg·g^-^¹, and 74.70889 mg·g^-^¹ respectively (**Supplementary Table S7**), which it was significant differences compared with other genotypes. These values indicate a greater tendency to increase Mg and Ca utilization and enhance, suggesting TT as the superior genotype. For 2D_29369331 and 3B_14919731 loci, materials with the genotypes AA and AG respectively, had average phenotypic effect values of 7.19463 mg·g^-^¹ and 7.70677 mg·g^-^¹, all greater than 0 and representing the highest positive effects (**Supplementary Table S7**). These results suggest a strong tendency for Mn utilization, thereby proposing the two genotypes as superior. Therefore, the three corresponding superior genotypes (TT, AA and AG) of these three SNPs can serve as important marker loci for the breeding of nutrient-efficient sugarcane varieties solidly.

Based on these three SNP marker loci, we screened materials with genotypes and high nutrient efficiency. The results showed that when using the possession of the TT excellent genotype at 3B_33126337 as the screening condition (**Supplementary Table S8**), there were a total of 23 materials with magnesium content higher than the average value of 267.34549 mg·g^-^¹; 25 materials with calcium content higher than the average value of 760.34603 mg·g^-^¹, while 22 materials (M142-59, Co281, Chuan-83-181, CP72-1210, yue91_976, Fu_nong-02-3924, HOCP03-704, Zhanzhe-45, Yunzhe-99-155, CP49-50, Co331, YCE07-74, HOCP07-612, GT-03-1403, GT-05-3084, Liucheng-05-291, YCE01-105, HOCP02-623, YCE07-65, Yunzhe-89-151, Co740, PS01, and Zhanzhe-80-101) had magnesium and calcium content higher than the average values of all materials.

Similarly, when using 2D_29369331 (AA) and 3B_14919731 (AG) as screening conditions respectively, the numbers of materials with manganese content higher than the average value of 13.63706 mg·g^-^¹ were 5 and 7 respectively, and finally totaling 12 materials that only possessed one manganese-related genotype was identified respectively (**Supplementary Table S8**). Noticing, the manganese content of C85-51(64.71526mg·g^-^¹) having the loci of 3B_14919731 (AG), is much higher than the remaining 11 accessions (**Supplementary Table S8**). Moreover, it is worth noting that although C89–51 did not possess the excellent genotype TT (at 3B_33126337), it had an excellent variant locus (T), being a heterozygote CT, and also showed high values in magnesium content (559.65554 mg·g^-^¹), calcium content (1617.42627 mg·g^-^¹). Therefore, C89–51 can be used as an important breeding material in terms of high nutrient efficiency. Besides, Zhanzhe-80-101 (magnesium: 635.24332 mg·g^-^¹, calcium:1894.98219 mg·g^-^¹, manganese: 29.12880 mg·g^-^¹), GT-05-3084 (722.02054 mg·g^-^¹, 1647.68226 mg·g^-^¹, 21.95156 mg·g^-^¹) and Zhanzhe-45 (646.84687 mg·g^-^¹, 1657.09416 mg·g^-^¹, 19.26045 mg·g^-^¹) contain TT (3B_33126337) and AA (2D_29369331) excellent genotype (**Supplementary Table S8**), and all showed better magnesium, calcium and manganese utilization.

In summary, based on the excellent genotypes identified in this study, gene editing technology can be used to precisely optimize important sugarcane materials in the future, or by formulating hybrid combinations (C89–51 and Zhanzhe-80–101 or GT-05–3084 or Zhanzhe-45) to aggregate the excellent genotypes of these materials, and breed materials will contain more high-quality loci (**Table 2**). These means will be conducive to promoting the breeding process of sugarcane with high nutrient efficiency.

**Table 2:** The information of high quality hybrid combination prediction.

P1	P2	P1-Superior genotype	P1-Superior genotype	Superior genotype of offspring
C89-51	Zhanzhe-80-101	1	2	3
C89-51	GT-05-3084	1	2	3
C89-51	Zhanzhe-45	1	2	3

P1 represents parent 1; P2 represents parent 2.

### 3.5 Molecular characteristics of candidate gene

Based on the linkage disequilibrium analysis, candidate genes of the three SNP markers (3B_33126337, 2D_29369331, 3B_14919731) were searched within the± 37Kb. A total of 12 candidate genes (**Supplementary Table S9**) were identified, which were related to magnesium, calcium, and manganese elements, respectively. The STRING software was used to perform interaction analysis on the proteins they expressed. The results showed that, except for Sspon.002D0013570, the other 11 proteins could be recognized by the database. However, the potential interaction relationships among them were weak (**Supplementary Figure S4**). Only with the Sspon.002D0013550 protein as the core was there a potential interaction with Sspon.002D0013560, Sspon.003B0007030, and Sspon.003B0007060 at a low confidence (0.15) level. Notably, Sspon.002D0013570 was also not found to have homologous proteins in other species in the NCBI database (https://blast.ncbi.nlm.nih.gov/). Only in the phytozome database (https://phytozome-next.jgi.doe.gov/) was it found to have a 10.77% similarity (**Supplementary Figure S5**) with Misin05G155400.1.p protein (Transferrin receptor-like, dimerization domain) of *Miscanthus sinensis*. This indicates that Sspon.002D0013570 is specifically selected for sugarcane and may have a potentially unique function.

**Figure 5:**
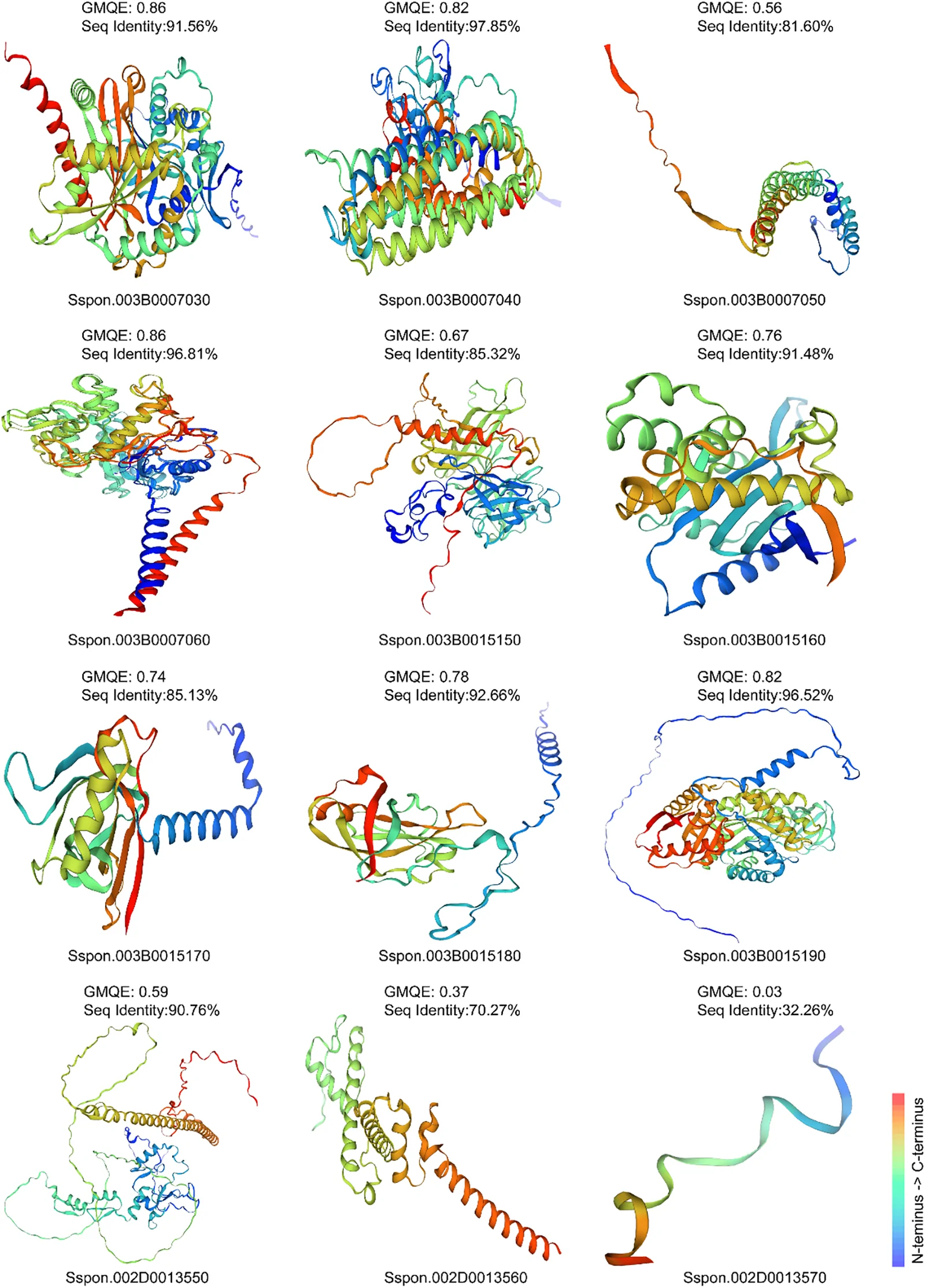
The three-dimensional structure of proteins. Predict the three-dimensional structure of proteins based on the SWISS-MODEL software. GMQE, namely Global model quality estimation, ranges from 0 to 1, and the higher the value is, the higher the reliability of the model will be. Seq identity refers to sequence homology.

In addition, the SWISS-model revealed significant differences in the three-dimensional structures among the 12 proteins (**Figure 5**), which in addition to Sspon.002D0013570, their established 3 D structures have high reliability (GMQE> 0.5) and good sequence match (seq identity> 70%), so the results have reference value. Then, their subcellular localizations were predicted, and they were found to potentially localize in various subcellular structures (**Supplementary Table S10**), such as chloroplasts, mitochondria, nuclei, endoplasmic reticula, plasma membrane, vacuolar, and even the cytoplasm. These suggest that the regulatory mechanisms of nutrient elements such as magnesium, calcium, and manganese might be complex and diverse.

Furthermore, to understand the molecular characteristics of these proteins, the number of amino acids, molecular weights, isoelectric points, and hydrophilicities were calculated (**Supplementary Table S11**). The results showed that the number of amino acids ranged from 133 aa to 576 aa, and the molecular weights ranged from 27,907.12 Da to 99,484.60 Da, with a wide distribution, indicating potential functional diversity. The isoelectric point values ranged from 4.92 to 10.52. Four of the proteins had isoelectric points less than 7, suggesting that the positively charged groups on their surfaces were deprotonated in an alkaline environment, rendering them negatively charged. The remaining eight proteins had isoelectric points greater than 7, indicating that the negatively charged groups on their surfaces would be protonated in an acidic environment, making them positively charged. The hydrophilicity values ranged from - 1.05 to 0.11. Nine proteins had hydrophilicity values less than 0, indicating a larger number of hydrophobic amino acids with greater contributions. For example, the protein Sspon.002D0013560 had the highest hydrophobicity value (- 1.05), suggesting that it might be involved in functions such as membrane structure support, transmembrane transport, or signal transduction in protein-protein interactions. Additionally, the remaining three proteins exhibited hydrophilicity. The protein Sspon.003B0007060 had the highest hydrophilicity (0.11), indicating a greater contribution of polar amino acids. It may promote the solubility of these proteins, act as a carrier for substance transport, participate in metabolic reactions, maintain the stability of intracellular and extracellular environments, and recognize hydrophilic signal molecules for signal transduction.

### 3.6 Functions prediction and RT-qPCR

To further understand the functions of the proteins, the types of protein families to which they belonged were predicted. The results showed that eight proteins were related to 23 protein families (**Supplementary Figure S6**, **Supplementary Table S12**). The protein Sspon.003B0015190 was predicted to have 13 conserved protein family domains, suggesting diverse functions. The proteins Sspon.003B000706, Sspon.003B001518, and Sspon.003B001516 were each predicted to have two conserved protein family domains. The proteins Sspon.003B0007040, Sspon.003B000705, Ssp0n.003B0015150, and Sspon.003B0007030 were classified into one protein family. However, none of these proteins had the same conserved protein family domain, and four proteins were not found to have conserved structures and were not classified into any protein family, further indicating significant differences in their structures and potentially diverse functional characteristics.

Moreover, to further identify the functions of these proteins, GO and KEGG analyses were performed. The GO analysis (P < 0.05) identified 22 GO terms (**Supplementary Table S13**), including 11 biological process, 8 molecular function, and 3 cellular component. Notably, the candidate protein Sspon.003B0015190 related to magnesium content (**Supplementary Table S9**), located in chloroplasts with identity = 66.96% (**Supplementary Table S10**), was found to belong to the molecular_function of magnesium ion binding (GO:0000287) in the GO analysis, further validating the reliability of this study. The KEGG analysis (P < 0.05) identified three pathways (**Supplementary Table S13**), namely Pyruvate metabolism (ath00620), Purine metabolism (ath00230), and Glycolysis/Gluconeogenesis (ath00010), and the normal operation of these pathways requires the utilization of mineral nutrient elements such as magnesium, calcium, and manganese.

To understand the expression patterns of these 12 candidate genes, we predicted the cis acting elements upstream of their +1 region by 2000bp, and found that all 12 genes had abundant functional elements (**Supplementary Figure S7**; **Supplementary Table S14**), including seed-specific regulation, the anaerobic induction, light responsiveness, defense and stress responsiveness, low-temperature responsiveness, the abscisic acid responsiveness, meristem expression, drought-inducibility, the Methyl Jasmonate responsiveness, zein metabolism regulation, auxin responsiveness, circadian control, gibberellin-responsiveness, salicylic acid responsiveness, and cell cycle regulation. Among them, an important transcription factor MYB has been identified (**Supplementary Table S14**), and up to 8 genes (*Sspon. 002D0013550*, *Sspon.003B0007030*, *Sspon.003B0007040*, *Sspon. 003B0007060*, *Sspon.003B00015160*, *Sspon.003B00015170*, *Sspon. 003B00015180*, *Sspon. 003B00015190*) have been found to have MYB binding sites in their upstream promoter regions, which can serve as an important theoretical basis for subsequent research on gene transcription regulation.

**Figure 6:**
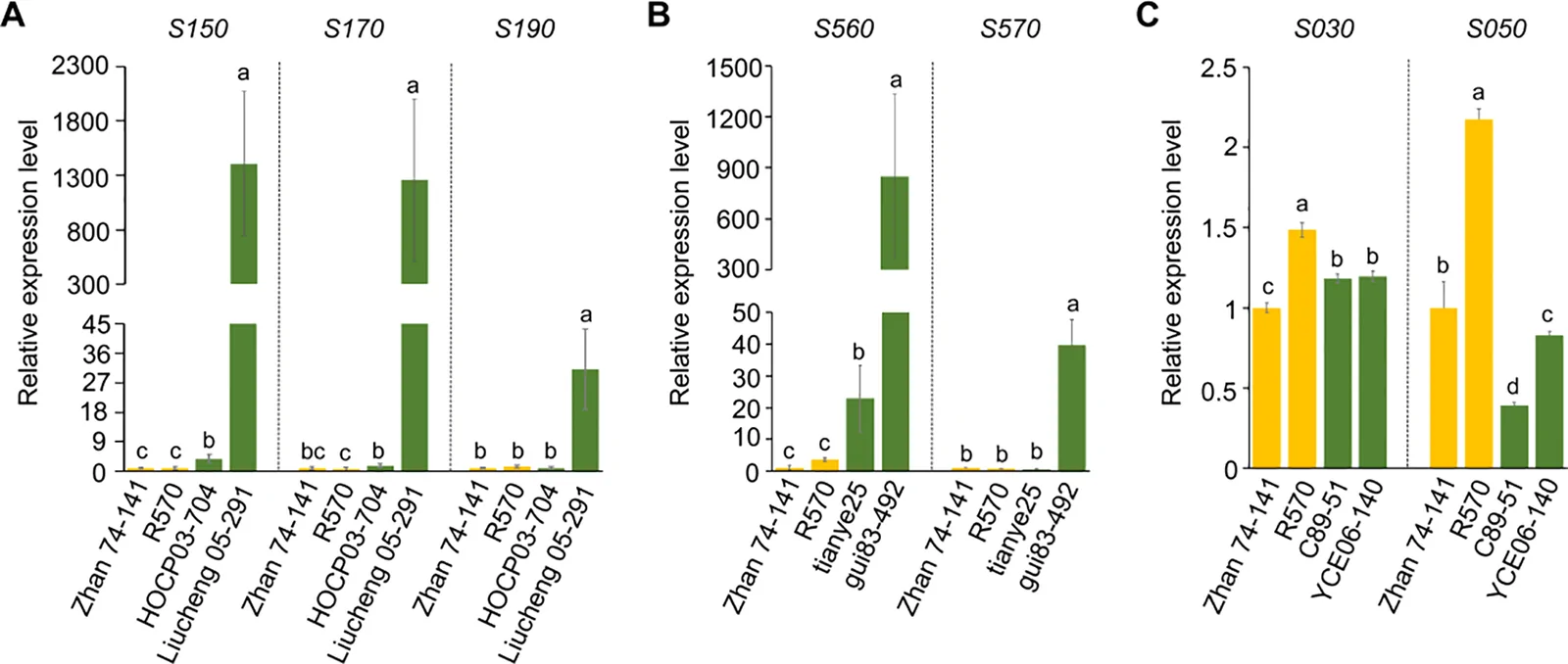
RT-qPCR Results of Candidate Genes. **(A)** The relative expression levels of candidate genes associated with Peak SNP 3B_33126337 among materials with or without the excellent genotype TT at this locus. **(B)** The relative expression levels of candidate genes associated with Peak SNP 2D_29369331 among materials with or without the excellent genotype AA at this locus. **(C)** The relative expression levels of candidate genes associated with Peak SNP 3B_14919731 among materials with or without the excellent genotype AG at this locus. Zhan74–141 was used as the control, and *GADPH* was employed as the internal reference. Materials without the excellent genotypes of the above three loci were regarded as the control group materials (Zhan74–141 and R570), and the columns representing them are colored yellow. Materials containing only the excellent genotype of the corresponding locus and without the other two excellent genotypes were used as the experimental group materials, namely HOCP03–704 and Liucheng-05-291, tianye25 and Gui-83-492, and C89–51 and YCE06-140, and the columns representing them were colored green. *S150*, *S170*, *S190*, *S560*, *S570*, *S030* and *S050* represent the candidate genes *Sspon.003B0015150*, *Sspon.003B0015170*, *Sspon.003B0015190*, *Sspon.002D0013560*, *Sspon.002D0013570*, *Sspon.003B0007030*, *Sspon.003B0007050*, respectively. Triplicate experiments were conducted, and variance analysis was performed to identify differences. Different letters indicate significant differences (P-value < 0.05).

It is worth mentioning that based on the CDS sequences of the homologous genes of 12 candidate genes in sugarcane, RT-qPCR primer design was carried out for 7 candidate genes with highly specific sequences (**Supplementary Table S15**). Among them, the candidate genes linked to the 3B_33126337 locus include *Sspon.003B0015150*, *Sspon.003B0015170*, and *Sspon.003B0015190*, while those of 2D_29369331 include *Sspon.002D0013560* and *Sspon.002D0013570*, and those of 3B_14919731 include *Sspon.003B0007030* and *Sspon.003B0007050*. Subsequently, materials containing only the excellent genotype TT (3B_33126337), namely HOCP03–704 and Liucheng-05-291, materials containing only the excellent genotype AA (2D_29369331), namely tianye25 and Gui-83-492, and materials containing only the excellent genotype AG (3B_14919731), namely C89–51 and YCE06-140, were selected, which each material did not contain the other two excellent genotypes as experimental group materials. Additionally, control materials that did not contain these three excellent genotypes, namely, zhan74–141 and R570, were selected. In total, 8 materials (**Supplementary Table S16**) were chosen for RT-qPCR. The results showed that for the experimental group materials containing only the excellent genotypes TT (3B_33126337) and AA (2D_29369331), the relative expression levels of the *Sspon.003B0015150* gene (**Figure 6A**) and the *Sspon.002D0013560* gene (**Figure 6B**) were significantly higher than those of the control materials. Meanwhile, for the experimental group materials containing the excellent genotype AG (3B_14919731), the relative expression level of the *Sspon.003B0007030* gene (**Figure 6C**) was significantly lower than that of the control materials, and all these results showed consistency. However, the relative expression levels of the other four genes did not show a linear relationship between the experimental group and the control group materials (**Figures 6A-C**). Consequently, *Sspon.003B0015150*, *Sspon.002D0013560*, and *Sspon.003B0007030* are suggested as genes worthy of significant attention, and their further functions can be deeply explored in subsequent studies.

## 4 Discussion

Nutrient elements are essential for the well-being of both plants and animals. These nutrients enter the human food chain through plants (Huang et al., 2020). Therefore, optimizing the phenotype of nutrient elements in plants is beneficial for human nutritional health. Sugarcane, especially its juice, is a crucial source of sugar and is widely used in food processing, holding great significance for human health. Hence, this study focuses on the optimization of nutrient elements in sugarcane. Here, we analyzed the contents of 14 macro - and micro - elements in the juice of 109 sugarcane samples. We found that all these elements exhibited certain variations, with most of their distributions approximating a normal distribution. There were also some materials showing extreme variations. For instance, the coefficient of variation of molybdenum exceeded 80%, indicating that the selected association population has high genetic diversity. This is conducive to the subsequent discovery of key loci and the screening of potential high - quality materials.

More importantly, we discovered that three pairs of elements, namely iron and total sugar, zinc and potassium, and molybdenum and manganese, showed good linear relationships, which is consistent with the research results of (Ma et al., 2023) However, the correlations between some elements presented opposite trends. For example, the Pearson correlation between calcium and phosphorus was 0.250* and the partial correlation analysis showed - 0.215*. The Pearson correlation between manganese and calcium was 0.340** and the partial correlation analysis showed a negative correlation of - 0.230*. These results suggest that the absorption and utilization of different elements by plants are complex and influenced by multiple factors, which is in line with the findings of (Freeland-Graves and Lin, 1988). Notably, through breeding value analysis, an important candidate material, C89 - 51, was identified. It is rich in 14 nutrient elements and has high breeding value, making it a suitable chassis material for subsequent variety breeding. This result is consistent with the findings of (Sandhu et al., 2022), who reported the identification of high - yielding and nutrient - efficient sugarcane lines based on BLUP values. Nevertheless, the different rankings of C89–51 in macronutrient and micronutrient breeding values indicate that the genetic factors governing nutrient accumulation vary for different nutrients, further emphasizing the need for a targeted breeding approach.

Discovering important gene loci and optimizing materials at the genetic level is an important approach. Therefore, based on SNP genotyping data, we confirmed that the selected materials possess abundant genetic diversity. For example, the kinship coefficients between the 109 sugarcane samples are relatively low. Specifically, 99.58% of the combinations have kinship coefficients less than 0.5, indicating that most of the materials have weak or no kinship. In addition, the average R² value of 0.0388 indicates a low linkage disequilibrium (LD) between SNPs. The LD decay distance of approximately 37 kb suggests that genetic recombination occurs over relatively short distances. This pattern of LD decay is consistent with the findings of (Tripathy, 2022), who observed similar low LD values and short decay distances in sugarcane, suggesting high genetic diversity and independent assortment of alleles. This implies that genetic markers can be effectively associated with traits without being confounded by strong LD, enabling more accurate identification of loci involved in key agronomic traits. Furthermore, based on population structure analysis, the materials can be classified into three subgroups. Compared with the classification by (Zhao et al., 2022), who only divided the materials into two subgroups based on genotyping results, the larger number of subgroups in our study further demonstrates the relatively rich genetic diversity of the selected population. More importantly, principal component analysis (PCA) also classified the materials into three subgroups, which is consistent with the results of population structure analysis. This indicates that the coefficients used to correct for population stratification are reliable and can reduce false positives (Mezmouk et al., 2011). All these results suggest that the selected association population is conducive to subsequent association analysis.

The use of both population structure correction coefficients (Q values and PCA values) and kinship coefficients as covariates is a powerful approach to reduce the occurrence of false-positive associations, as it effectively accounts for the potential confounding effects of population structure and relatedness among individuals (Sul et al., 2018). Hence, we used the above three correction factors to execute MLM_Q + Kinship and MLM_PCA + Kinship model. The result showed a high degree of consistency, with lambda values near 1, indicating that the association models were reliable (Lai et al., 2024). Notably, the two models identified a similar set of SNP loci for each nutrient element and their breeding values, with a total of 59 reliable “Peak SNPs” being identified under the most stringent conditions (P-value < 1.53×10^-6^). These SNPs explained more than 29% of the phenotypic variance for specific traits, indicating that they have substantial genetic effects. The identification of such loci is crucial for breeding programs aimed at improving nutrient content in sugarcane, as these SNPs can serve as markers for selecting varieties with optimal nutrient profiles.

The identification of SNP loci associated with macronutrients such as magnesium and calcium (SNP locus 3B_33126337) and micronutrients like iron (SNP locus 5C_64683842) is particularly noteworthy. The SNP locus 3B_33126337, which was identified in both breeding values (8-BLUP and 14-BLUP), is likely to play a critical role in the uptake and utilization of these key nutrients. Similarly, the SNP locus 5C_64683842, identified for its association with iron content, and especially related to micronutrient breeding values (6-BLUP), may offer insights into the genetic basis of micronutrient efficiency in sugarcane. These findings are consistent with previous studies, such as (Dhanapal et al., 2018), which reported specific loci associated with nutrient uptake efficiency in crops, including magnesium and calcium. Furthermore, the discovery of SNPs that explain a significant proportion of the phenotypic variance for these traits adds to the growing body of knowledge on genetic regulation of nutrient composition in crops. Previous GWAS studies on nutrient elements in crops such as rice and maize have similarly identified key loci that influence mineral content, which are being used to improve nutrient quality in these species (Ma et al., 2021). The ability to pinpoint such loci in sugarcane is a critical step toward the development of biofortified varieties, which can provide more nutritious food sources and contribute to addressing malnutrition, particularly in developing regions where sugarcane is a staple crop.

More importantly, the phenotypic effects of 59 reliable SNP loci on nutrient content in sugarcane were thoroughly analyzed to identify superior genotypes and screen for nutrient-efficient candidates in this study. The results indicate that certain genotypes at specific SNP loci are associated with higher absorption and accumulation of key nutrients such as magnesium (Mg), calcium (Ca), and manganese (Mn), which are essential for improving the nutritional profile of sugarcane. The identification of these superior genotypes opens up exciting opportunities for breeding nutrient-efficient sugarcane varieties. The SNP locus 3B_33126337, associated with Mg and Ca, emerged as particularly important. The TT genotype at this locus showed significantly higher phenotypic values for Mg and Ca (26.09966 mg·g^-^¹ for Mg and 74.70889 mg·g^-^¹ for Ca), highlighting its role in enhancing nutrient utilization. This finding is consistent with studies on other crops, where specific loci have been linked to improved nutrient uptake (Alcock et al., 2017). Therefore, the TT genotype at 3B_33126337 is considered a superior genotype, suggesting that individuals possessing this allele could be prioritized in breeding programs aimed at improving Mg and Ca content in sugarcane. Similarly, for the SNP loci 2D_29369331 and 3B_14919731, associated with Mn, the AA and AG genotypes, respectively, showed higher positive phenotypic effect values (7.19463 mg·g^-^¹ and 7.70677 mg·g^-^¹). These genotypes were also found to exhibit a strong tendency for Mn utilization, making them valuable targets for breeding nutrient-efficient sugarcane varieties. The identification of these superior genotypes provides new insights into the genetic basis of Mn uptake, a critical micronutrient for plant growth.

Subsequently, the screening of materials based on these superior genotypes revealed several sugarcane accessions with high nutrient efficiency. Valuably, C89-51, which did not possess the ideal TT genotype at 3B_33126337, still demonstrated high nutrient content, especially for magnesium and calcium, due to its heterozygous CT genotype. This highlights the importance of considering both homozygous and heterozygous variants when selecting superior traits, as heterozygosity may still contribute significantly to nutrient accumulation. Furthermore, accessions such as Zhanzhe-80-101, GT-05-3084, and Zhanzhe-45 exhibited excellent nutrient profiles, suggesting that they are strong candidates for breeding programs focused on improving nutrient efficiency in sugarcane. The findings from this study align with the growing body of research that seeks to optimize nutrient content in crops through the identification of key genetic markers. Previous studies in other crops, such as rice and maize, have similarly identified loci associated with mineral nutrient content, and the results of this study further extend this knowledge to sugarcane (Bohar et al., 2020). The identification of superior genotypes for Mg, Ca, and Mn utilization in sugarcane represents a step forward in developing varieties that are not only more nutritious but also more resilient to nutrient deficiencies in the soil.

Besides, understanding the functions of these genes is beneficial for further optimization at the genetic level, thereby fundamentally promoting the breeding of nutritionally efficient varieties. Hence, 12 candidate genes associated with the SNP markers 3B_33126337, 2D_29369331, and 3B_14919731, were identified respectively that linked to magnesium (Mg), calcium (Ca), and manganese (Mn) uptake in sugarcane. One particularly interesting result is the identification of *Sspon.002D0013570*, a gene with no homologous proteins in other species and only given very low similarity between Sspon.002D0013570 and Misin05G155400.1.p in Miscanthus sinensis, which could point to a sugarcane-specific mechanism. Similar unique genes have been identified in other crop such as sorghum (Thirugnanasambandam et al., 2018; Baloch et al., 2023).

Then, given bioinformatics analysis, these 12 candidates proteins are scarcely related, exhibit significant differences in molecular characteristics, have a wide distribution of potential subcellular localizations, distinct three - dimensional structures, and belong to protein families with obvious disparities. However, promoter element analysis showed that they respond to various environmental factors such as light and drought, highlighting the complexity of the regulatory network involved in nutrient transport (Wang et al., 2019). Particularly, here identified that there are MYB binding sites in the promoter regions of several genes, which MYB transcription factors have been reported to regulate nutrient uptake and stress tolerance in crops like maize and rice (Yuan et al., 2021; Wu et al., 2024). Excitingly, Gene Ontology (GO) analysis showed that Sspon.003B0015190, related to magnesium, was classified as having the molecular function of magnesium ion binding, directly supporting its role in magnesium uptake. Similar functional classifications were reported in other plants, like maize where ZmMTP6, involved in Mg transport, is categorized as a magnesium ion - binding protein (Zhao et al., 2024). KEGG pathway analysis revealed candidate genes’ involvement in metabolic pathways such as pyruvate, purine, and glycolysis/gluconeogenesis. These require magnesium, calcium, and manganese, and are crucial for energy production and cellular function. The participation of nutrient - related genes in these pathways indicates a complex nutrient regulation system beyond simple ion transport. The role of these pathways in plant metabolism and stress response is well - studied in other plants. For example, Mg in Arabidopsis’ glycolysis and pyruvate metabolism are vital for optimal growth and stress response (Aoki - Kinoshita and Kanehisa, 2007). Identifying these pathways in sugarcane emphasizes that efficient nutrient uptake is essential for growth, stress resilience, and overall plant health. All these results enhance the reliability of our findings.

Finally, to further determine the usability of some candidate genes, we conducted an alignment of the homologous sequences of 12 candidate genes in sugarcane. It was found that specific sequence loci for primer design could only be identified in 7 candidate genes, indicating the differentiation specificity of these 7 candidate genes. The other 5 genes had highly homologous sequences at other positions on the sugarcane chromosomes, but no related SNP markers were associated with them. This implies that the 7 relatively specific candidate genes, which are respectively linked to the three identified loci (3B_33126337, 2D_29369331, 3B_14919731) may specifically influence the nutrient absorption function of sugarcane. Therefore, RT - qPCR analysis was performed on these 7 relatively specific candidate genes. Fortunately, based on the three loci, we identified an important candidate gene for each locus, namely *Sspon.003B0015150* (associated with locus 3B_33126337), *Sspon.002D0013560* (2D_29369331), and *Sspon.003B0007030* (3B_14919731). This indicates that these three candidate genes are highly potential candidates affecting nutrients such as magnesium, calcium, and manganese in sugarcane.

In summary, this study highlights the significant phenotypic effects of specific SNP loci on nutrient content in sugarcane and provides valuable insights for breeding nutrient-efficient varieties. By focusing on superior genotypes identified for Mg, Ca, and Mn, breeders can improve the nutritional profile of sugarcane, which could have far-reaching implications for global food security and public health. The integration of molecular breeding techniques, such as gene editing and hybridization, with these findings promises to advance the development of high-yield, nutrient-rich sugarcane cultivars. For example, gene-editing technologies have been successfully applied in the multi-crops to improve zinc uptake through the manipulation of nutrient transporters (Kumar et al., 2024). Similarly, the identification of the 3B_33126337 SNP locus associated with magnesium and calcium content in sugarcane offers a promising target for future gene-editing strategies aimed at improving nutrient efficiency. The identification of C89-51, which, despite not possessing the ideal genotype at 3B_33126337, still showed high nutrient efficiency, demonstrates the potential of using a combination of favorable alleles to enhance nutrient efficiency, as suggested by other studies in maize and wheat (Nagamine and Ezura, 2022).

## 5 Conclusion

**Figure 7:**
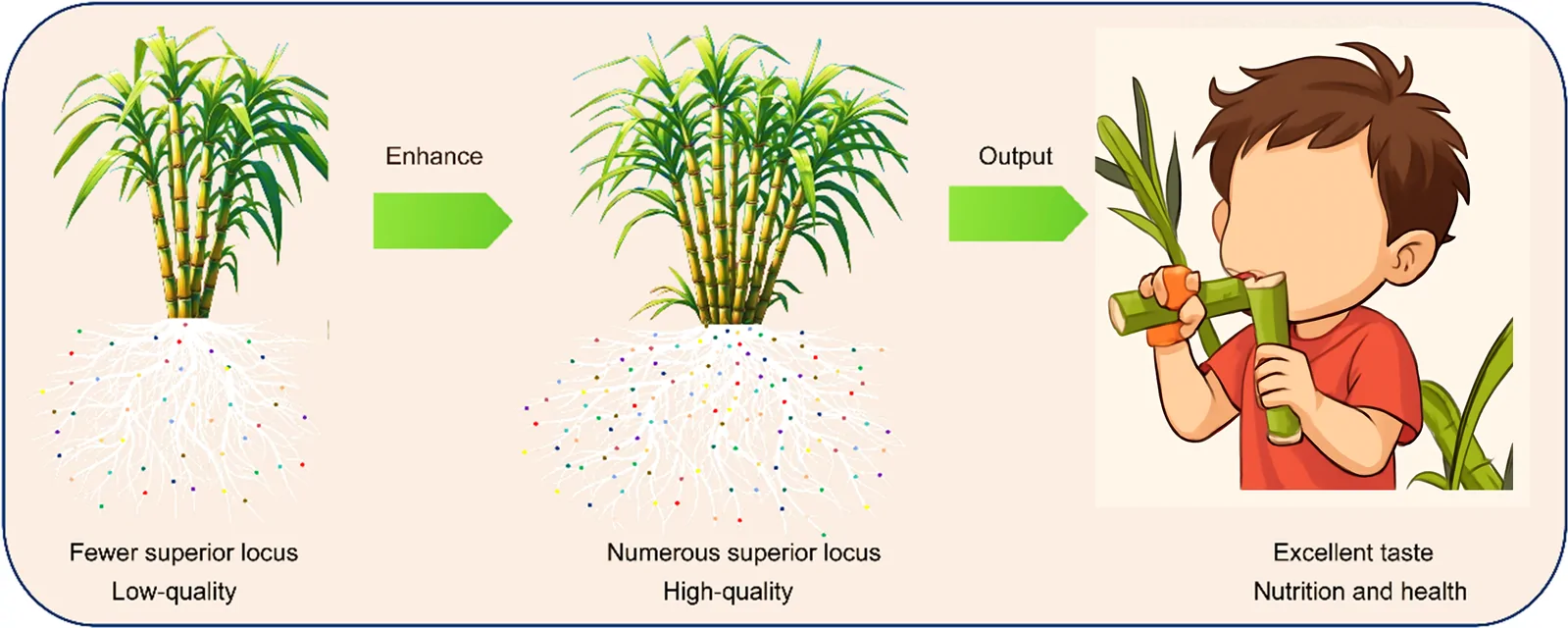
The route for cultivating tasty, nutritious and healthy sugarcane. Discover the gene loci related to high nutrient efficiency in sugarcane and optimize the combination of superior genotypes based on them. This aims to incorporate more favorable loci, resulting in high yield, good taste, rich nutrition, and better health benefits for humans. Eventually, achieve the “Genetic dissection of nutrient element enrichment in *Saccharum officinarum* L. for deciphering food health.

Based on GWAS, we have identified some peak SNP marker association with nutrient element. Importantly, after rigorous screening of multiple conditions, three more important SNP marker and three excellent genotypes were identified. Based on these results, we discovered 12 candidate genes. Through bioinformatic analysis, we obtained a more detailed understanding of the molecular features and potential functions of these 12 candidate genes. Especially three of the candidate genes (*Sspon.003B0015150*, *Sspon.002D0013560*, and *Sspon.003B0007030*), were found to show significant differences in the relative expression level detection between materials with or without excellent genotypes and thus could be given key attention. These results can provide directions for further functional verification in subsequent studies. More importantly, utilizing the findings of this research to improve sugarcane varieties with a few excellent loci that result in low quality will contribute to the breeding of sugarcane varieties that aggregate more excellent loci and possess high quality. Ultimately, it will accelerate the breeding process of sugarcane varieties with high yield, good nutrition, and excellent taste (**Figure 7**), and promote the security of global sugar production as well as ensure the nutrition and health of food.

## Data Availability

The original contributions presented in the study are included in the article/**Supplementary Material**. Further inquiries can be directed to the corresponding authors.

## Supplementary material

Supplementary Figure 1 The distribution of macronutrient elements. **(A-H)** The distribution of TS, N, P, K, S, Mg, Ca, Si elements respectively. ZT, N, P, K, S, Mg, Ca, and Si represent total sugar, nitrogen, phosphorus, potassium, sulfur, magnesium, calcium, and silicon, respectively.

Supplementary Figure 2 The distribution of micronutrient elements. **(A-F)** The distribution of Fe, Mn, Zn, Cu, Mo, Ge elements respectively. Fe, Mn, Zn, Cu, Mo, Ge represents iron, manganese, zinc, copper, molybdenum, and Germanium, respectively.

Supplementary Figure 3 Manhattan and Q-Q plot. Manhattan plot and Q-Q plot of **(A)** GWAS results based on MLM-Q+Kinship model. Manhattan plot and Q-Q plot of **(C)** GWAS results are based on MLM-PCA+Kinship model. ZT, N, P, K, S, Mg, Ca, Si, Fe, Mn, Zn, Cu, Mo, and Ge represent total sugar, nitrogen, phosphorus, potassium, sulfur, magnesium, calcium, silicon, iron, manganese, zinc, copper, molybdenum, and germanium, respectively. λ-value represents Lambda value, and the closer it is to 1, the more reliable the model is.

Supplementary Figure 4 Protein-protein interaction analysis. The interaction between proteins was predicted using the STRING software. Spheres of different colors represent individual proteins, with the type of protein labeled in the upper right corner of each sphere. Lines connecting the spheres indicate interactions between proteins, and the thicker the line, the stronger the potential interaction.

Supplementary Figure 5 Comparison diagram of amino acid sequences. Based on phytozome database( https://phytozome-next.jgi.doe.gov/)Identified protein MisIn05G155400.1. P with weak similarity to Sspon.00200013570.

Supplementary Figure 6 Promoter elements upstream of the gene +1 region within 2000bp. Different colors represent different functions of components, while their length represents the length of the sequence involved in the component.
