# Correction: Genetic dissection of nutrient element enrichment in *Saccharum officinarum* L. for deciphering the food health

**DOI:** 10.3389/fpls.2026.1994020

**Published:** 2026-09-21

**Authors:** Lina Fan, Zaid Chachar, Ming Chen, Yucong Li, Jiarui Chen, Huiyi He, Jiakun Wen, Saira Gul, Ruiqiang Lai, Yongwen Qi

**Affiliations:** 1 College of Agriculture and Biology, Zhongkai University of Agriculture and Engineering, Guangzhou, Guangdong, China; 2 Institute of Nanfan and Seed Industry, Guangdong Academy of Science, Guangzhou, Guangdong, China; 3 Shenzhen Wemed Medical Equipment Co., Ltd., Shenzhen, China

**Keywords:** *Saccharum officinarum*, nutrient element, GWAS, genetic dissection, food health

An incorrect **Funding** statement was provided. The original statement read:

“The author(s) declare financial support was received for the research and/or publication of this article. The project was supported by the National Natural Science Foundation of China (32072027), The Guangdong Province special projects in key fields of ordinary colleges and universities, the Guangdong Province key construction discipline research ability enhancement project (2022ZDJS023), Key Research Project of Guangdong Provincial Department of Education (2022ZDZX4017), Guangzhou Science and Technology Plan Project (SL2023A04J02318), The Special Project for Rural Revitalization Strategy in Guangdong Province (2022-NPY-00-023-5).”

The correct **Funding** statement reads:

“The author(s) declare that financial support was received for the research and/or publication of this article. The project was supported by the National Natural Science Foundation of China (32072027), The Guangdong Province Special Projects in Key Fields of Ordinary Colleges and Universities, the Guangdong Province Key Construction Discipline Research Ability Enhancement Project (2022ZDJS023), Key Research Project of Guangdong Provincial Department of Education (2022ZDZX4017), Guangzhou Science and Technology Plan Project (SL2023A04J02318), and the 2024 Guangdong Rural Revitalization Strategy Special Fund Seed Industry Project (First Batch) (Yue Nong Nong Ji [2024] No. 102).”

The original version of this article has been updated.

