# Supplementary material for: Genetic dissection of nutrient element enrichment in Saccharum officinarum L. for deciphering the food health
Source: Front Plant Sci. 2025 Dec 4;16:1649792. doi: 10.3389/fpls.2025.1649792 (PMC12711768; doi:10.3389/fpls.2025.1649792)
Supplement: Supplementary Figure 1 — The distribution of macronutrient elements. (A-H) The distribution of TS, N, P, K, S, Mg, Ca, Si elements respectively. ZT, N, P, K, S, Mg, Ca, and Si represent total sugar, nitrogen, phosphorus, potassium, sulfur, magnesium, calcium, and silicon, respectively. [file Supplementaryfile1.docx]

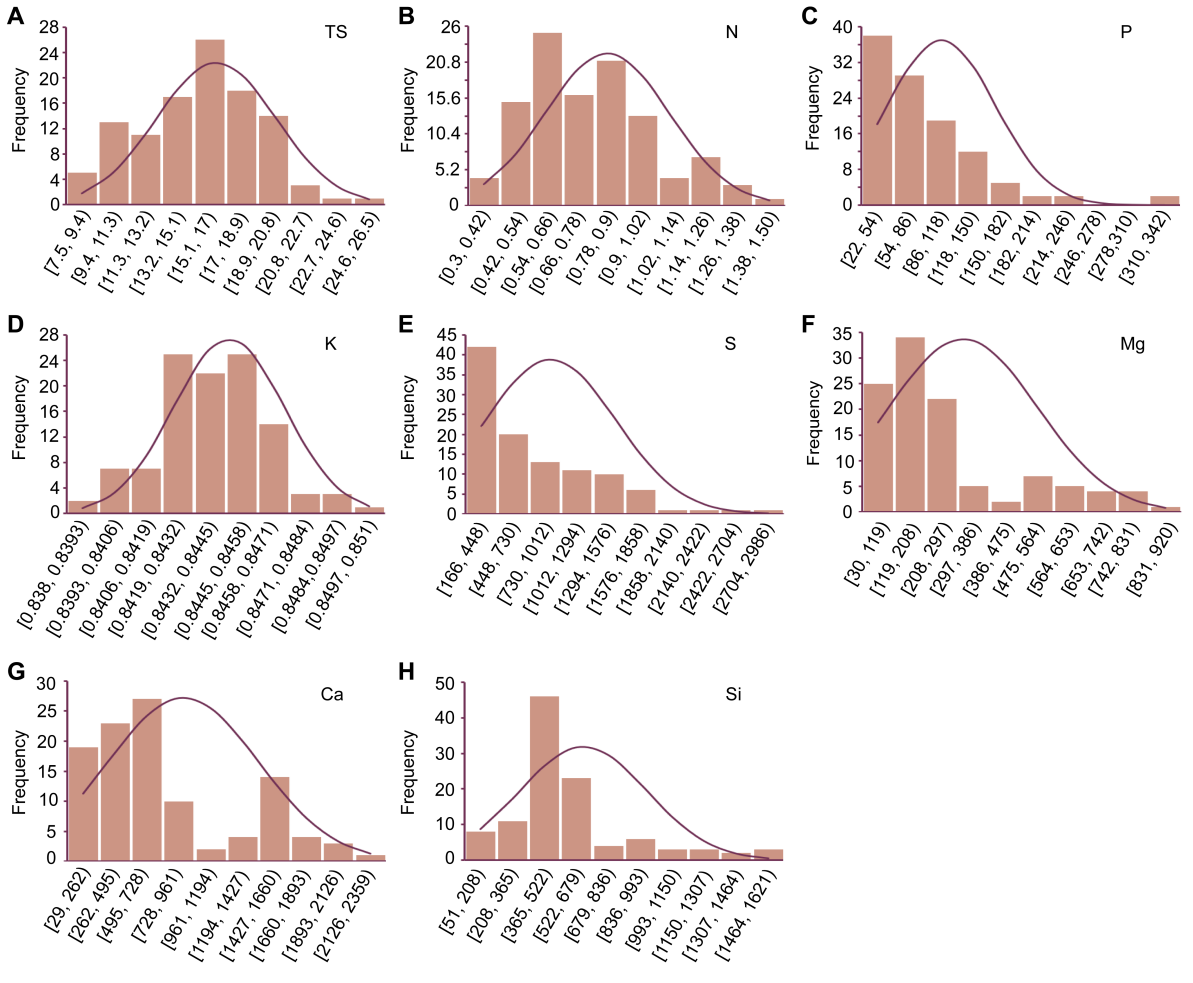


**Figure S1.** The distribution of macronutrient elements. (A-H) The distribution of TS, N, P, K, S, Mg, Ca, Si elements respectively. ZT, N, P, K, S, Mg, Ca, and Si represent total sugar, nitrogen, phosphorus, potassium, sulfur, magnesium, calcium, and silicon, respectively.


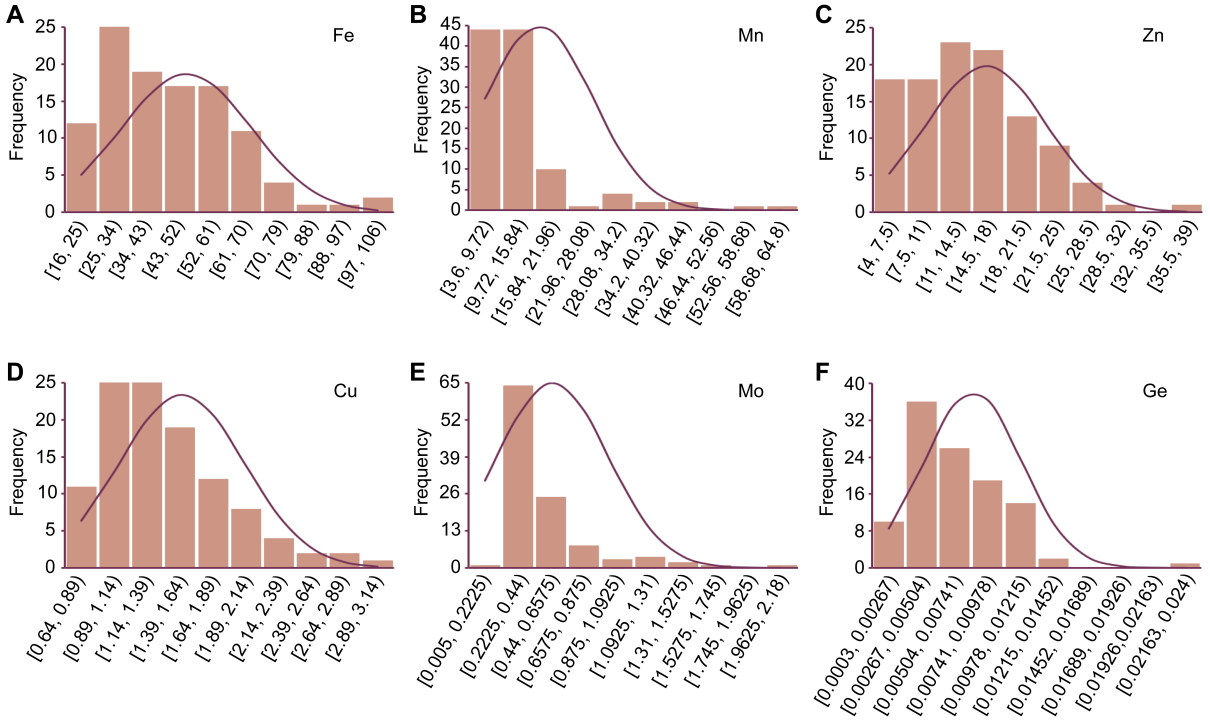


**Figure S2.** The distribution of micronutrient elements. (A-F) The distribution of Fe, Mn, Zn, Cu, Mo, Ge elements respectively. Fe, Mn, Zn, Cu, Mo, Ge represents iron, manganese, zinc, copper, molybdenum, and Germanium, respectively.


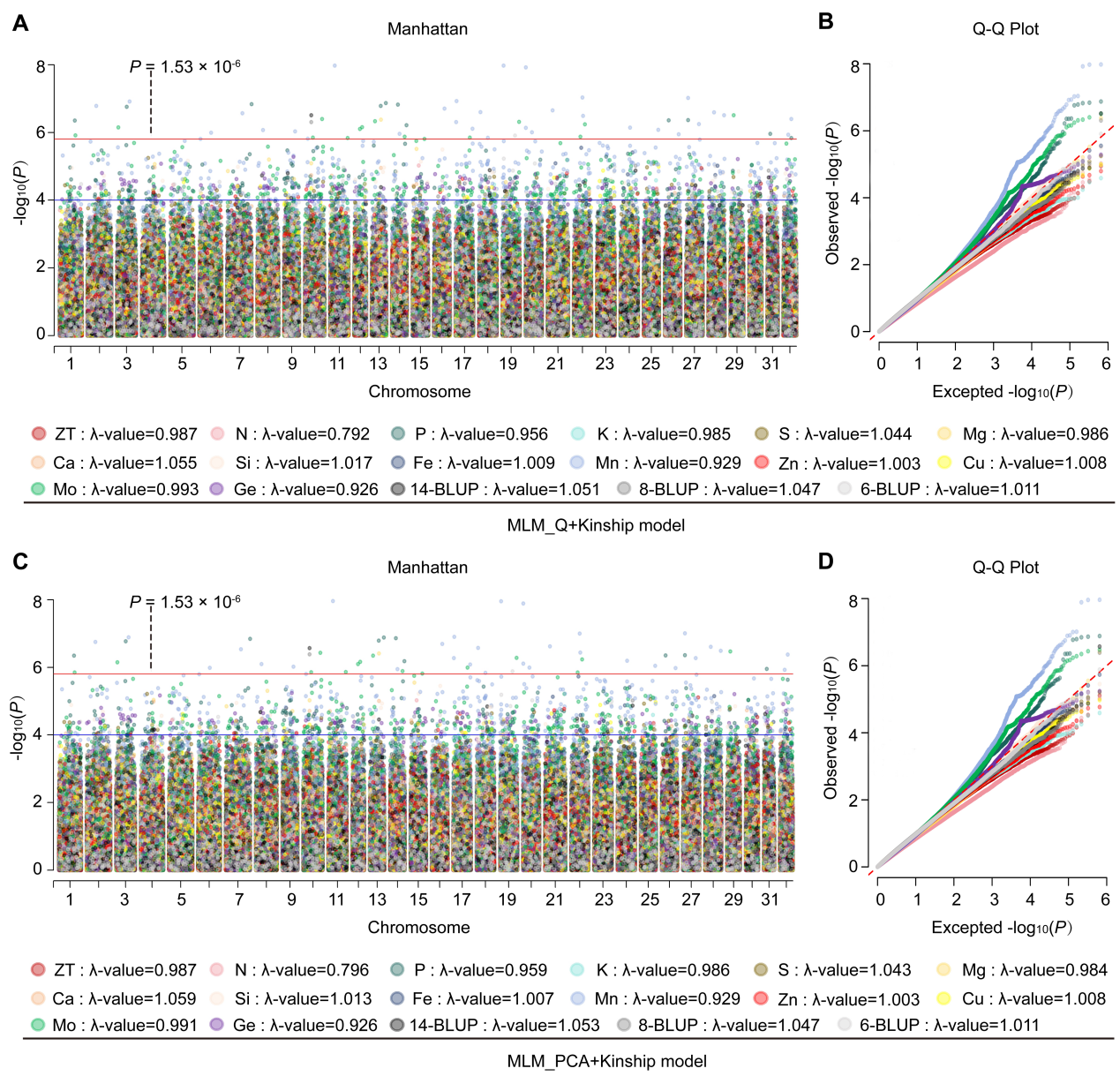


**Figure S3**. Manhattan and Q-Q plot. Manhattan plot and Q-Q plot of (A) GWAS results based on MLM-Q+Kinship model. Manhattan plot and Q-Q plot of (C) GWAS results are based on MLM-PCA+Kinship model. ZT, N, P, K, S, Mg, Ca, Si, Fe, Mn, Zn, Cu, Mo, and Ge represent total sugar, nitrogen, phosphorus, potassium, sulfur, magnesium, calcium, silicon, iron, manganese, zinc, copper, molybdenum, and germanium, respectively. λ-value represents Lambda value, and the closer it is to 1, the more reliable the model is.


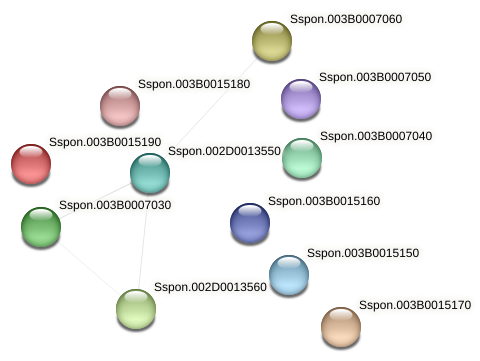


**Figure S4. Protein-Protein Interaction Analysis.** The interaction between proteins was predicted using the STRING software. Spheres of different colors represent individual proteins, with the type of protein labeled in the upper right corner of each sphere. Lines connecting the spheres indicate interactions between proteins, and the thicker the line, the stronger the potential interaction.


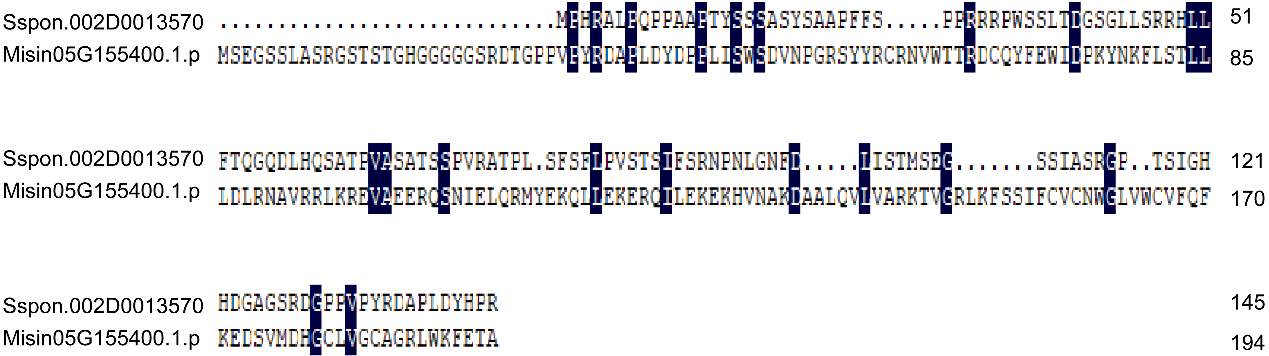


Figure S5 Comparison diagram of amino acid sequences. Based on phytozome database（ https://phytozome-next.jgi.doe.gov/ ）Identified protein MisIn05G155400.1. P with weak similarity to Sspon.00200013570


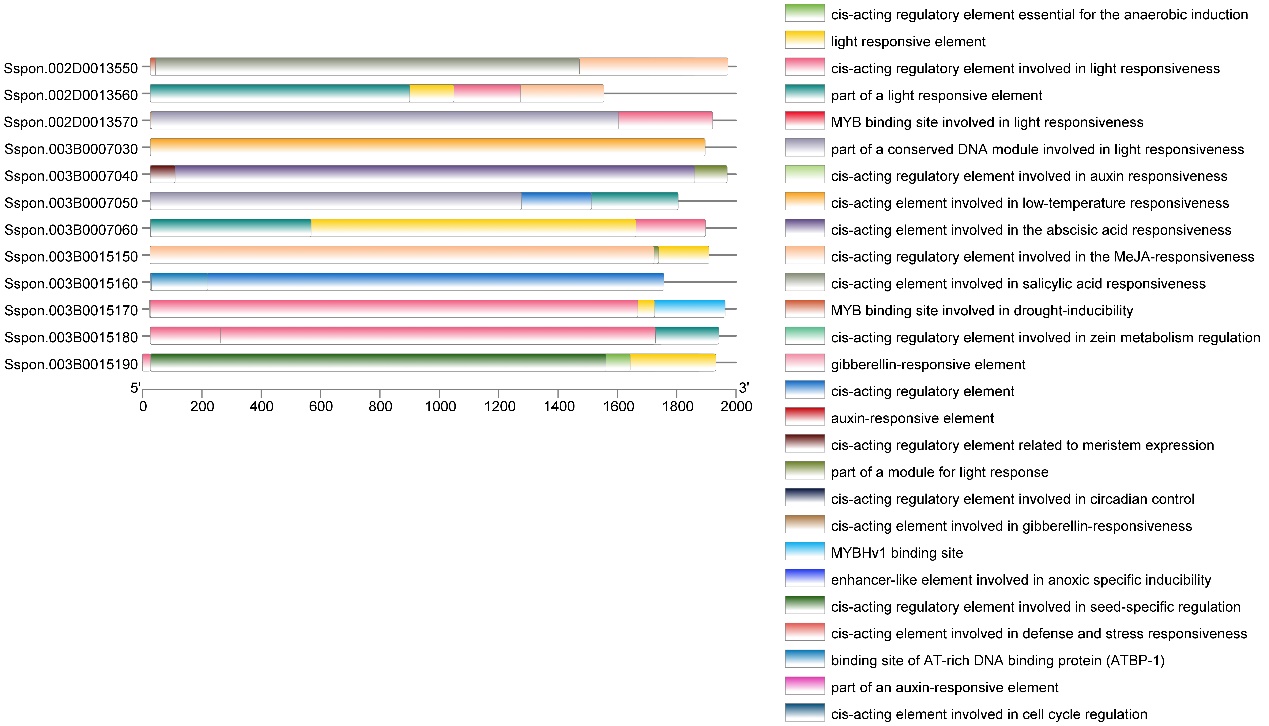


**Figure S6**. Promoter elements upstream of the gene +1 region within 2000bp. Different colors represent different functions of components, while their length represents the length of the sequence involved in the component.
